# Balancing functions of antifouling, nitric oxide release and vascular cell selectivity for enhanced endothelialization of assembled multilayers

**DOI:** 10.1093/rb/rbae096

**Published:** 2024-08-24

**Authors:** Sulei Zhang, Jun Sun, Shuaihang Guo, Yichen Wang, Yuheng Zhang, Jiao Lei, Xiaoli Liu, Hong Chen

**Affiliations:** State and Local Joint Engineering Laboratory for Novel Functional Polymeric Materials, College of Chemistry, Chemical Engineering and Materials Science, Soochow University, Suzhou215123, P. R. China; The SIP Biointerface Engineering Research Institute, Suzhou215123, P. R. China; State and Local Joint Engineering Laboratory for Novel Functional Polymeric Materials, College of Chemistry, Chemical Engineering and Materials Science, Soochow University, Suzhou215123, P. R. China; State and Local Joint Engineering Laboratory for Novel Functional Polymeric Materials, College of Chemistry, Chemical Engineering and Materials Science, Soochow University, Suzhou215123, P. R. China; State and Local Joint Engineering Laboratory for Novel Functional Polymeric Materials, College of Chemistry, Chemical Engineering and Materials Science, Soochow University, Suzhou215123, P. R. China; State and Local Joint Engineering Laboratory for Novel Functional Polymeric Materials, College of Chemistry, Chemical Engineering and Materials Science, Soochow University, Suzhou215123, P. R. China; State and Local Joint Engineering Laboratory for Novel Functional Polymeric Materials, College of Chemistry, Chemical Engineering and Materials Science, Soochow University, Suzhou215123, P. R. China; State and Local Joint Engineering Laboratory for Novel Functional Polymeric Materials, College of Chemistry, Chemical Engineering and Materials Science, Soochow University, Suzhou215123, P. R. China; The SIP Biointerface Engineering Research Institute, Suzhou215123, P. R. China

**Keywords:** surface modification, layer-by-layer self-assembly, nitric oxide release, heparin analogs, endothelialization

## Abstract

Surface endothelialization is a promising way to improve the hemocompatibility of biomaterials. However, current surface endothelialization strategies have limitations. For example, various surface functions are not well balanced, leading to undesirable results, especially when multiple functional components are introduced. In this work, a multifunctional surface was constructed by balancing the functions of antifouling, nitric oxide (NO) release and endothelial cell promotion via layer-by-layer (LBL) self-assembly. Poly(sodium *p*-styrenesulfonate-*co-*oligo(ethylene glycol) methacrylate) (negatively charged) and polyethyleneimine (positively charged) were deposited on silicon substrates to construct multilayers by LBL self-assembly. Then, organic selenium, which has a NO-releasing function, and the cell-adhesive peptide Gly-Arg-Glu-Asp-Val-Tyr, which selectively promotes endothelial cells, were introduced on the assembled multilayers. Poly(oligo(ethylene glycol) methacrylate) is a hydrophilic component for antifouling properties, and poly(sodium *p*-styrenesulfonate) is a heparin analog that provides negative charges. By modulating the contents of poly(oligo(ethylene glycol) methacrylate) and poly(sodium *p-*styrenesulfonate) in the copolymers, the NO release rates catalyzed by the modified surfaces were regulated. Moreover, the behaviors of endothelial cells and smooth muscle cells on modified surfaces were well controlled. The optimized surface strongly promoted endothelial cells and inhibited smooth muscle cells to achieve endothelialization effectively.

## Graphical abstract

**Figure:**
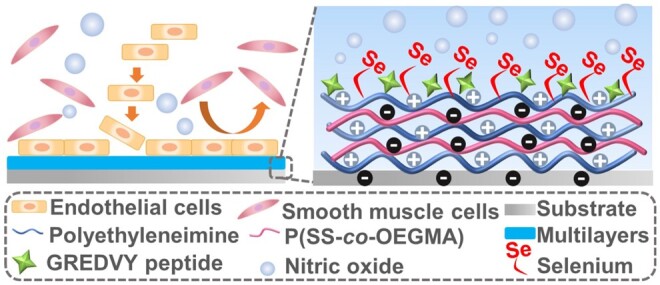

## Introduction

In blood vessels, there is an intact layer of endothelial cells in contact with flowing blood. A healthy and intact endothelial monolayer protects against inflammatory reactions and clotting [1]. When blood vessels are damaged, the integrity of the endothelium is disrupted, leading to excessive proliferation and migration of smooth muscle cells, triggering neointimal proliferation and delaying endothelialization [2]. Achieving endothelialization to inhibit hyperplasia is the ideal situation for long-term implants such as vascular stents or vascular grafts.

Many strategies, such as building hydrophilic surfaces, building bioactive surfaces and building surfaces with topological structures, have been developed to promote endothelialization on surfaces [2–4]. Hydrophilic polymers and immunosuppressive agents were incorporated on surfaces to inhibit smooth muscle cells, but they also inhibited the adhesion and proliferation of endothelial cells [5–9]. Surfaces modified with cell-adhesive peptides [3, 10–12] and heparin analogs [13, 14] promoted the growth of endothelial cells, but these surfaces did not effectively inhibit smooth muscle cells. It is difficult to achieve endothelialization using a single surface modification strategy, but preparing multifunctional surfaces by multiple strategies is possible.

Many studies have focused on the construction of multifunctional surfaces to promote endothelialization. For example, cell-adhesive peptides were combined with hydrophilic polymers to regulate the migration of endothelial cells and smooth muscle cells [15]. Nitric oxide (NO) produced by healthy endothelial cells was reported to act in a dose-dependent manner, with lower concentrations of NO generally promoting cell adhesion and proliferation, while higher concentrations lead to cell cytotoxicity [16]. Co-immobilization of NO-releasing molecules with other functional molecules on surfaces is an important way to promote endothelialization [17–20]. For example, hydrophilic polymers and selenocystamine (a catalyst for the release of NO) were co-immobilized on the surface, and sulfonate groups were subsequently introduced through host–guest interactions. The obtained surface was able to promote endothelial cells while inhibiting smooth muscle cells [21]. The difficulty in building multifunctional surfaces lies in introducing multiple functional molecules by a simple method and balancing the conflicts among different functions. Layer-by-layer (LBL) self-assembly, a powerful method for introducing multiple functional molecules to surfaces with great simplicity and universality, is the ideal surface modification strategy for solving these problems [22].

Herein, a multifunctional endothelialization surface was constructed by combining hydrophilic polymers, heparin analogs, organic selenium and cell-adhesive peptides via LBL self-assembly. First, a series of copolymers, poly(sodium *p*-styrenesulfonate-*co*-oligoethylene glycol methacrylate), with different chemical compositions were synthesized by modulating the feed ratio of the monomers sodium *p*-styrenesulfonate (SS) and oligo(ethylene glycol) methacrylate (OEGMA). The hydrophilic component poly(oligo(ethylene glycol) methacrylate) (POEGMA) was designed to provide an antifouling background. The heparin analog poly(sodium *p*-styrenesulfonate) (PSS) was designed to promote cells and supply negative charges to the copolymers. Polyethyleneimine (PEI) is a positively charged polyelectrolyte that contains a large number of amino groups that can be used to graft organic selenium. Copolymers and PEI were deposited on silicon (Si) substrates by LBL self-assembly, after which selenocystamine was grafted on the multilayers to introduce NO release function. Finally, the peptide Gly-Arg-Glu-Asp-Val-Tyr (GREDVY) was introduced as the top layer on the surface to selectively promote endothelial cells. By modulating the contents of heparin analogs and hydrophilic components in copolymers, multifunctional surfaces capable of balancing endothelial cell promotion and smooth muscle cell inhibition to achieve endothelialization were obtained.

## Experimental section

### Materials

Single crystal silicon wafers ((100)-oriented) were purchased from Guangzhou Semiconductor Materials (Guangzhou, China). The as-received wafers were polished on one side and diced into square chips of 0.5 cm × 0.5 cm in size. 2,2′-azoisobutyronitrile (98%), acetonitrile (99%), *N, N'-*dimethylformamide (99%), triethylamine (99.5%), *N, N'*-carbonyl diimidazole (98%), selenocystamine dihydrochloride (SeCA, 97%), S-nitrosoglutathione (GSNO, 98%) and OEGMA (*M*_n_ = 500 g/mol, 99%) were purchased from Aladdin. SS (95%), paraformaldehyde, Triton X-100, and PEI (*M*_n_ = 2.5 × 10^4^ g/mol, 95%) were purchased from Sigma-Aldrich. A NO Griess Assay Kit was purchased from Beyotime (Shanghai). L-glutathione (GSH, 98%) and ethylenediaminetetraacetic acid (EDTA) were purchased from Macklin. GREDVY (98%) peptide was from GL Biochem Ltd (Shanghai). Actin-Tracker Green (phalloidin-FITC), 4',6-diamidino-2-phenylindole (DAPI) were purchased from Solarbio. Calcein acetoxymethyl ester (calcein-AM) and propidium iodide (PI) were purchased from Dojindo. A human cyclic guanosine monophosphate (cGMP) enzyme-linked immunosorbent assay kit was purchased from Meibiao Biology. RIPA lysis buffer, anti-GAPDH rabbit, anti-alpha smooth muscle actin rabbit, anti-CD31 rabbit and horseradish peroxidase conjugated goat anti-rabbit IgG (H+L) were purchased from Servicebio. Human umbilical vein vascular endothelial cells (HUVECs), human umbilical vein vascular smooth muscle cells (HUVSMCs), endothelial cell culture medium (ECM), and smooth muscle cell culture medium (SMCM) were purchased from MeisenCTCC. 5-Chloromethylfluorescein diacetate (Cell-Tracker Green CMFDA) and 5-(and-6)-(((4-chloromethyl) benzoyl)amino) tetramethyl rhodamine) (Cell-Tracker Orange CMTMR) were purchased from MKbio. Solvents were purified before use.

### Synthesis of copolymers with different chemical compositions

Copolymers of SS and OEGMA were synthesized by free-radical polymerization. SS and OEGMA at different monomer feed ratios were dissolved in 10 ml of *N*,*N'*-dimethylformamide with 2,2′-azoisobutyronitrile (0.2 mg, 1.2 µmol) as the initiator. After 24 h of reaction at 65°C, the resulting mixture was dialyzed (3500 Da) and freeze-dried to obtain copolymers. To modulate the chemical compositions of the copolymers, four different monomer feed ratios were used. The OEGMA molar concentrations among the monomers were 4.76%, 9.09%, 13.04% and 16.67%. The corresponding copolymers were designated as PSO_n_ (*n* = 1, 2, 3, 4). For example, PSO_1_ was synthesized from the monomers SS (1 g, 4.85 mmol) and OEGMA (121 mg, 0.24 mmol).

### Preparation of PEI/PSO_n_ multilayers

Si substrates were treated with piranha solution and immersed in aqueous PEI (2 mg/ml) and PSO_n_ (2 mg/ml) for 5 min alternatively. Multilayers (7.5 bilayers) were constructed on Si by electrostatic interactions between positively charged PEI and negatively charged PSO_n_. The multilayers assembled on Si using different PSO_n_ copolymers were named PEI/PSO_n_.

### Incorporation of SeCA and GREDVY peptide into PEI/PSO_n_ multilayers

SeCA (2.4 mg, 0.0075 mmol) was dissolved in a mixture of 600 μl of acetonitrile and 240 μl of triethylamine. Then, 240 μl of acetonitrile containing *N, N'*-carbonyl diimidazole (2.5 mg, 0.015 mmol) was added to the mixture, which was subsequently activated for 4 h at 25°C. PEI/PSO_n_ samples were added to this activated solution and kept for 24 h at 25°C. *N*,*N'*-carbonyl diimidazole was used to couple amino groups of SeCA and PEI on the surface [23, 24]. After washing with acetonitrile and ultrapure water, the resulting samples were named PEI/PSO_n_-Se.

To incorporate the GREDVY peptide on the surface, PEI/PSO_n_-Se samples were immersed in a 1-mg/ml GREDVY aqueous solution (pH = 5) at 25°C for 1 h. After washing and vacuum drying overnight, PEI/PSO_n_-Se-G samples were obtained.

### Polymer and surface characterization

The copolymers and surfaces were characterized via Nicolet 6700 Fourier transform infrared spectroscopy (FTIR, Thermo Scientific Co., Inc.) and nuclear magnetic resonance spectroscopy (^1^H-NMR, Varian INOVA 400 MHz, Thermo Scientific Co., Inc.). The molecular weight (*M*_n_, Da) and polydispersity index (*Đ*) of the copolymers were characterized via gel permeation chromatography (GPC, Waters e3695, Polymer Lab Co., Inc.) with water containing 0.2 M NaNO_3_ and 0.1 M NaH_2_PO_4_ and adjusted to pH = 9 at 30°C (flow rate: 1 ml/min). GPC was calibrated based on the narrow molecular weight distribution of polyethylene glycol (PEG) standards [25]. The wettability of the different surfaces was characterized by an SL 200C optical contact angle meter (KINO Industry Co., Ltd). Surface morphology and roughness were characterized via atomic force microscopy (AFM, MultiMode8 SPM, Thermo Scientific Co., Inc.). The elemental composition of the different surfaces was characterized via X-ray photoelectron spectroscopy (XPS, ESCALAB 250 XI, Thermo Scientific Co., Inc.). The thickness of the multilayers was measured via a spectroscopic ellipsometer (M-2000V, J. A. Woollam Co., Inc.). The zeta potential was measured on a Nano-ZS model Zetasizer instrument (Nano-ZS90, Malvern, England). Q-Sense-E4 dissipative quartz crystal microscale (QCM-D, Sweden) was used to quantify the deposition of polymers and peptide. The absorbance of phosphate-buffered saline (PBS) solution was characterized by UV-Vis spectrophotometer (UV-3600, Shimadzu).

### *In vitro* catalytic generation of NO

The working solution for the NO release assay (65 μM GSNO, 30 μM GSH and 500 μM EDTA) was prepared first. The samples were placed in 5 ml of working solution and incubated at 37°C for 24 h. After incubation, a Griess Assay Kit was used to detect the amount of NO catalytically generated by the samples via ultraviolet-visible absorption spectroscopy (Varioskan^®^ Flash Multifunctional Enzyme Marker, Thermo Scientific Co., Inc.) as described previously [23].

### Adhesion and proliferation of HUVECs and HUVSMCs

HUVECs were cultured on samples (20 000 cells/cm^2^, 37°C, 5% CO_2_) for 24 and 72 h. NO donors (65 μM GSNO and 30 μM GSH) were added every 6 h during culture. After incubation, the cells were treated with 4% paraformaldehyde and 0.1% Triton X-100. Phalloidin-FITC was added to stain the cytoskeleton, and DAPI was used to stain the nuclei. Then, the cells were observed using an inverted fluorescence microscope (Olympus IX71 Carl Zeiss, Olympus Co., Inc.). The culture and staining of HUVSMCs were similar to those of HUVECs, and the medium used was SMCM.

### The expression level of cGMP in HUVSMCs

HUVSMCs were seeded on the samples at a density of 20 000/cm^2^ and cultured (37°C, 5% CO_2_) for 2 h with NO donors (65 μM GSNO and 30 μM GSH). After incubation, the HUVSMCs were treated with 10% Triton X-100 for 15 min and sonicated for 10 min. Then, the suspensions were centrifuged, and the cGMP concentrations in the HUVSMCs were determined as described previously [26].

### The expression level of α-SMA in HUVSMCs

HUVSMCs were seeded on the PEI/PSO_3_-Se-G samples at a density of 10 000 cells/cm^2^ and cultured (37°C, 5% CO_2_) for 72 h with NO donors (65 μM GSNO and 30 μM GSH). After incubation, the HUVSMCs were treated with RAPI lysis buffer on ice for 30 min. Then, the suspensions were centrifuged, and the expression level of α-SMA in HUVSMCs was determined by western blotting.

### The expression level of CD31 in HUVECs

HUVECs were cultured on Si and PEI/PSO_3_-Se-G samples (20 000 cells/cm^2^, 37°C, 5% CO_2_) for 24 and 72 h. NO donors (65 μM GSNO and 30 μM GSH) were added every 6 h during culture. After incubation, the cells were treated with 4% paraformaldehyde and 0.1% Triton X-100. Anti-CD31 rabbit and horseradish peroxidase conjugated goat anti-rabbit IgG (H + L) were added to stain the CD31 protein. DAPI was used to stain the nuclei. Then, the cells were observed using an inverted fluorescence microscope (Olympus IX71 Carl Zeiss, Olympus Co., Inc.).

### Migration of HUVECs

HUVECs were seeded on the samples at a density of 50 000 cells/cm^2^ and cultured (37°C, 5% CO_2_) until an intact cell monolayer was formed. A sterile 200 μl pipette tip was used to create scratches. After scratching, the HUVECs were stained with calcein-AM and observed by an inverted fluorescence microscope. HUVECs were cultured for 24 h after making scratches and NO donors (65 μM GSNO and 30 μM GSH) were added every 6 h during culture. After incubation, the HUVECs were observed by an inverted fluorescence microscope.

### Co-culture of HUVECs and HUVSMCs

HUVECs and HUVSMCs were first labeled with Green CMFDA and Orange CMTMR cell-trackers, respectively. Labeled cells were seeded on samples at a ratio of 1:1 (total density of 20 000/cm^2^) and cultured (37°C, 5% CO_2_) for 2 and 24 h. NO donors (65 μM GSNO and 30 μM GSH) were added every 6 h during culture. After incubation, a fluorescence microscope was used to observe the cells.

### Statistical analysis

Three parallel samples were used in each experiment, and all the experiments were repeated in triplicate. The data are expressed as the mean ± standard deviation. Significant differences between samples and Si were determined by the t test (*P <* *0.05, **P <* *0.01, ***P <* *0.001 and *P *>* *0.05 (n.s.)).

## Results and discussion

### Polymer characterization

**Figure 1.**
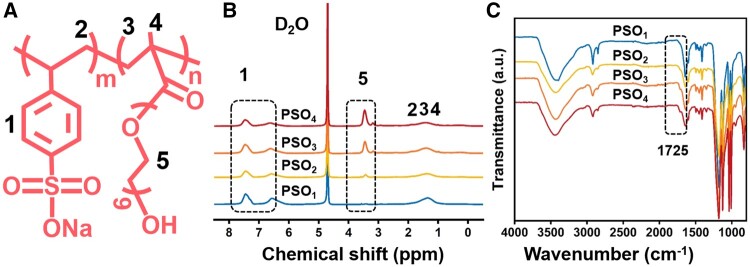
Chemical structure (**A**),^1^H-NMR spectra using heavy water (D_2_O) as the solvent (**B**) and FTIR spectra (**C**) of the PSO_n_ copolymers.

The molar concentrations of OEGMA among the monomers (*F*_OEGMA_/%) used for copolymerization are shown in Table 1. The chemical structure of the PSO_n_ copolymers is shown in Figure 1A. The characteristic peaks with a chemical shift of 3.0–3.6 ppm in ^1^H-NMR were attributed to methylene hydrogen in POEGMA (Figure 1B). The characteristic peaks with a chemical shift at 6.0–8.1 ppm belong to the proton peak of the benzene ring of the SS monomer unit. The contents of POEGMA and PSS in the PSO_n_ copolymers were calculated according to the integrated area of these two groups of characteristic proton peaks [27]. The contents of POEGMA in the copolymers calculated from ^1^H-NMR spectra (*f*_POEGMA_/%) were consistent with those of *F*_OEGMA_ for copolymerization. *f*_POEGMA_ levels in the copolymers increased with increasing *F*_OEGMA_ levels. As shown in Figure 1C, the absorption bands at 1184 and 1128 cm^−1^ in the FTIR spectra of the PSO_n_ copolymers were attributed to the stretching vibration of sulfonic acid groups (-SO_3_−) [28]. And the absorption band at 1725 cm^−1^ was attributed to the vibration of the carbonyl group in POEGMA [29]. The intensity of the absorption band at 1725 cm^−1^ increased with increasing *f*_POEGMA_ content. The conversion percentages ranged from 86% to 87% for the different copolymers. The GPC spectra of different copolymers are shown in Supplementary Figure S1. The molecular weights of the PSO_n_ copolymers synthesized with different feed ratios ranged from (1.30–1.40) ×10^4^ g/mol, and the *Đ* values of the copolymers ranged from 1.21 to 1.22 (Table 1). The PSO_n_ copolymers showed no significant differences in the molecular weights and *Đ* values.

**Table 1.** Molecular weights and chemical compositions of the different copolymers

Copolymers	*F*_OEGMA_ ^a^ (%)	*f*_POEGMA_ ^b^ (%)	*f*_PSS_ ^c^ (%)	*M*_n_ (g/mol)	*Đ*
PSO_1_	4.76	1.96	98.04	1.40 × 10^4^	1.21
PSO_2_	9.09	3.70	96.30	1.30 × 10^4^	1.22
PSO_3_	13.04	9.71	90.29	1.40 × 10^4^	1.21
PSO_4_	16.67	16.23	83.77	1.40 × 10^4^	1.21

a OEGMA molar concentrations among the monomers used to synthesize copolymers.

b,c Molar compositions of the copolymers determined by ^1^H-NMR.

### Preparation and characterization of PEI/PSO_n_ multilayers

**Scheme 1.**
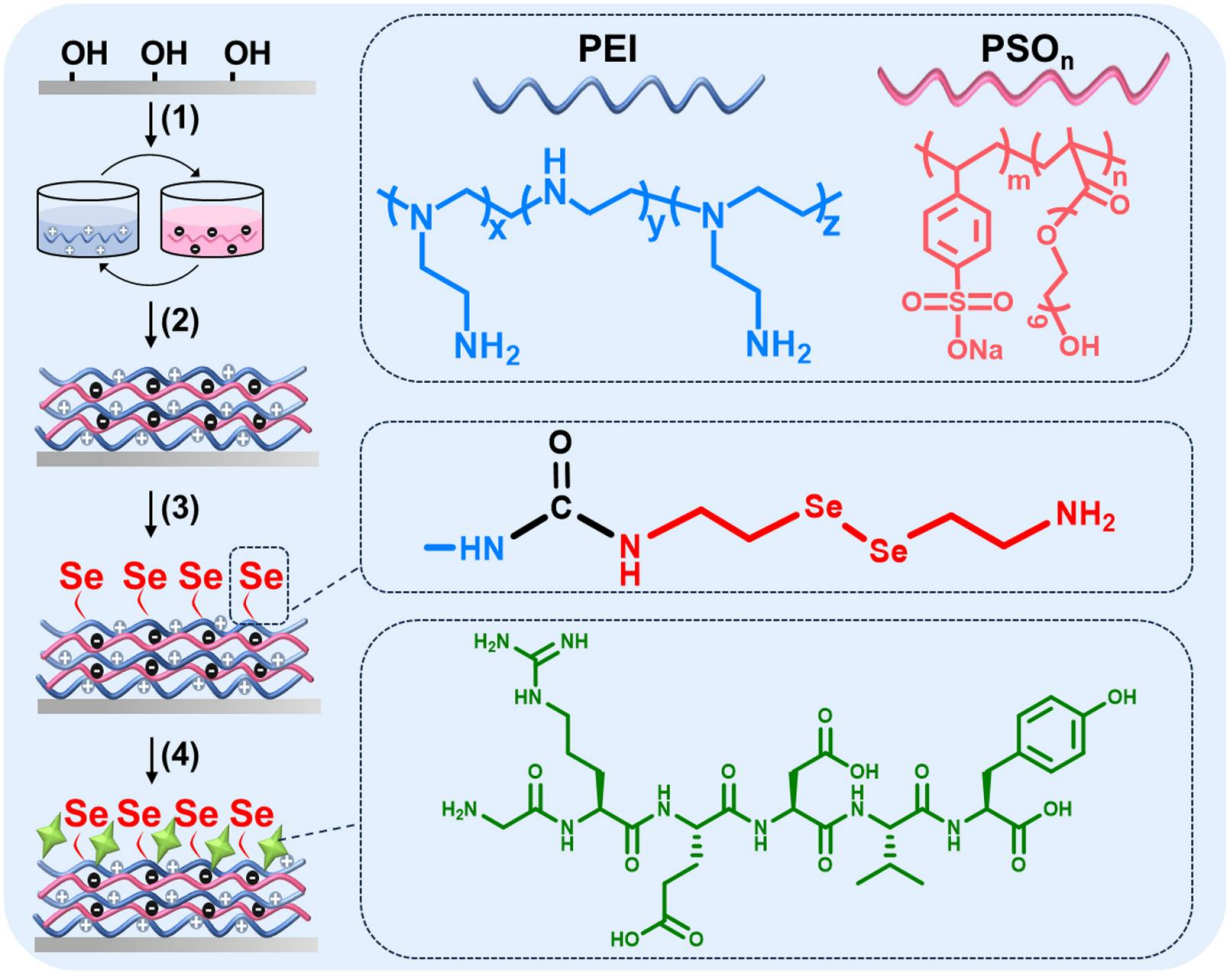
Construction of multifunctional surfaces. (1) and (2) PEI and PSO_n_ were deposited on piranha-treated Si to construct multilayers by LBL self-assembly. (3) SeCA was grafted on the multilayers. (4) The GREDVY peptide was immobilized on the surface as a top coating by electrostatic force.

Silicon wafers were selected as the model substrate for surface construction to facilitate the subsequent surface characterization. As shown in Scheme 1, positively charged PEI and negatively charged PSO_n_ were used to prepare PEI/PSO_n_ multilayers on Si by LBL self-assembly. The NO release rates and HUVEC density on surfaces with 5.5, 7.5 and 10.5 bilayers were studied (Supplementary Figure S2). It was determined that surfaces with 7.5 bilayers were the optimal configuration for further modification.

**Figure 2.**
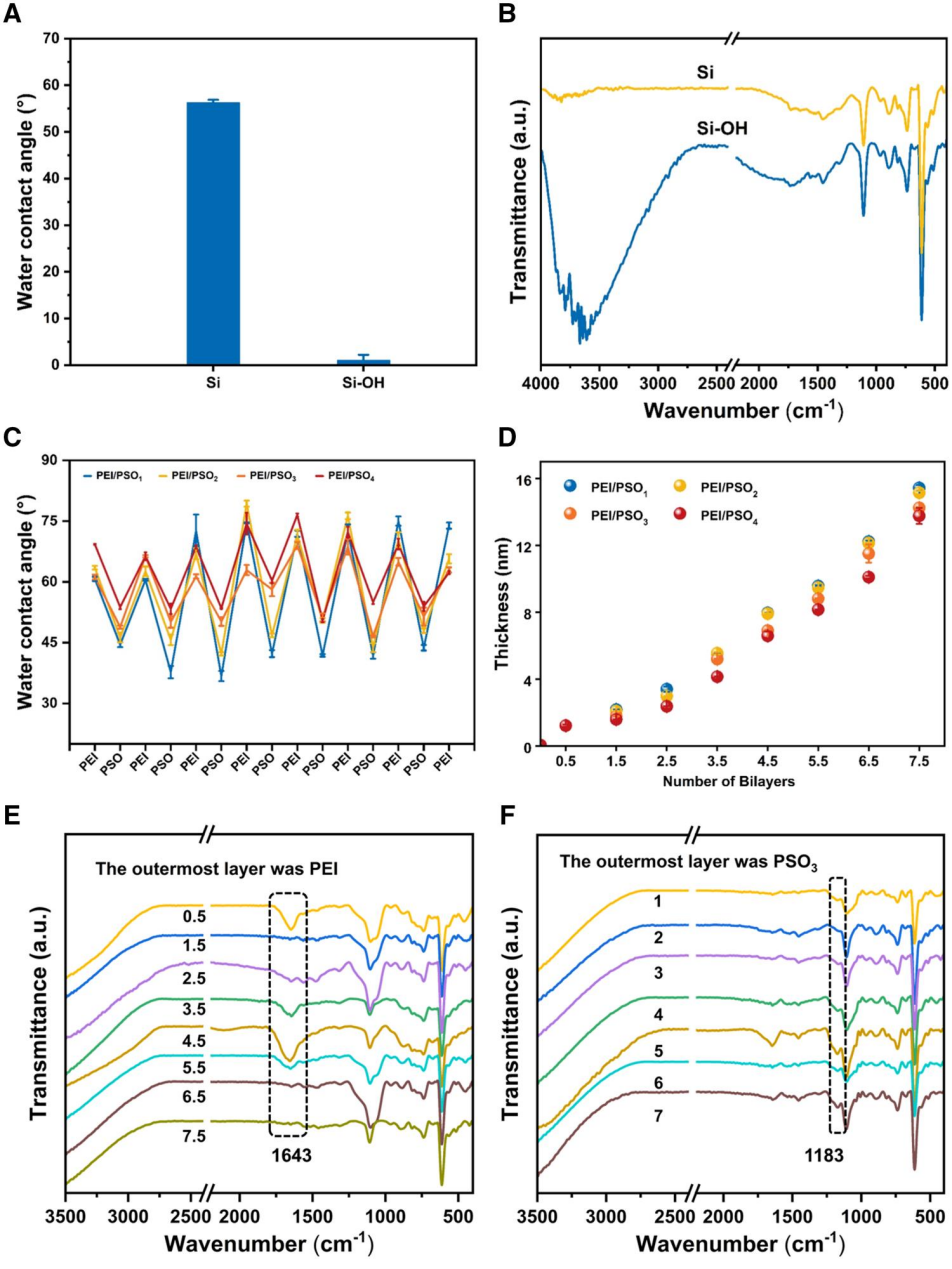
The water contact angle (**A**) and FTIR spectra (**B**) of Si and Si-OH surfaces. The water contact angle (**C**) and thickness (**D**) of multilayers during the LBL self-assembly process. FTIR spectra of surfaces with different numbers of bilayers. (**E**) The outermost layer was PEI. (**F**) The outermost layer was PSO_3_.

The modified surfaces were characterized by the static water contact angle, FTIR, ellipsometry, XPS and AFM. As shown in Figure 2A, the water contact angle of the Si surface was ∼60°. After treatment with piranha solution, the water contact angle of the Si-OH surface was ∼5°, indicating superhydrophilicity. In addition, a wide absorption band in the range of 3600–3400 cm^−1^ was observed on the Si-OH surface (Figure 2B), indicating the presence of hydroxyl groups [30]. As shown in Supplementary Table S1, the ratios of O/Si on the Si and Si-OH surfaces were 0.66 and 10.54, respectively. The significant increase in O/Si on the Si-OH surface compared to that on the Si surface indicated that a large number of hydroxyl groups appeared on the surface after the piranha solution treatment [11, 31]. As shown in Figure 2C, the water contact angle showed a sawtooth shape as the outermost polyelectrolyte of the multilayer changed as reported previously [32, 33]. When the outermost polyelectrolyte was PSO_n_, the water contact angle of the surfaces gradually increased with increasing *f*_POEGMA_ content in the copolymers. Previous studies have shown that the water contact angle was approximately 20°–30° for surfaces grafted with PSS [34, 35] and 50°–60° for surfaces grafted with POEGMA [36, 37]. It was therefore hypothesized that an increase in the amount of *f*_POEGMA_ would decrease the hydrophilicity of the PSO_n_-assembled surfaces. The thickness of the multilayers increased as the assembly process proceeded (Figure 2D).

When the number of the assembled bilayers was 0.5, 1.5, 2.5, 3.5, 4.5, 5.5, 6.5 and 7.5 with PEI as the outermost layers, an absorption peak at 1643 cm^−1^ was observed on the surface, which was attributed to the bending vibration of primary amine groups within the PEI structure [38] (Figure 2E). As shown in Figure 2F, when the number of the assembled bilayers was 1, 2, 3, 4, 5, 6 and 7 with PSO_3_ as the outermost layer, an absorption peak at 1183 cm^−1^ was observed on the surface, which was attributed to the sulfonic acid group in PSO_3_ [28]. The chemical compositions of the samples were further determined by XPS (Supplementary Table S2), with the process of LBL self-assembly, the chemical compositions of the surface changed. As shown in Supplementary Figure S3, SiO_2_ nanoparticles were used to measure the zeta potential after each layer coating. When the outermost layer was PEI, the particles were positively charged and the zeta potential was approximately 15 ∼50 mV. When the outermost layer was PSO_3_, the particles were negatively charged and the zeta potential was approximately −60∼−40 mV [39]. After constructing PEI/PSO_3_ multilayers on SiO_2_ chips, the mass of the chip increased by ∼9439 ng/cm^2^, as determined by QCM-D (Supplementary Table S3). These data indicate the successful assembly of PEI/PSO_n_ multilayers on the Si surface.

### Preparation and characterization of PEI/PSO_n_-Se-G

As shown in Scheme 1, SeCA was grafted on PEI/PSO_n_ multilayers using *N*,*N'*-carbonyl diimidazole as a coupling agent as reported previously[23, 24]. The GREDVY peptide consists of six amino acids with an isoelectric point of 3.6. The pH of the GREDVY aqueous solution was adjusted to 5 to make it negatively charged. Then, GREDVY peptide was introduced to the surface through electrostatic interactions between PEI and the peptide [40, 41]. After modifying the GREDVY peptide on the SiO_2_ chips, the mass of the chip increased by ∼766 ng/cm^2^ determined by QCM-D (Supplementary Figure S4).

**Figure 3.**
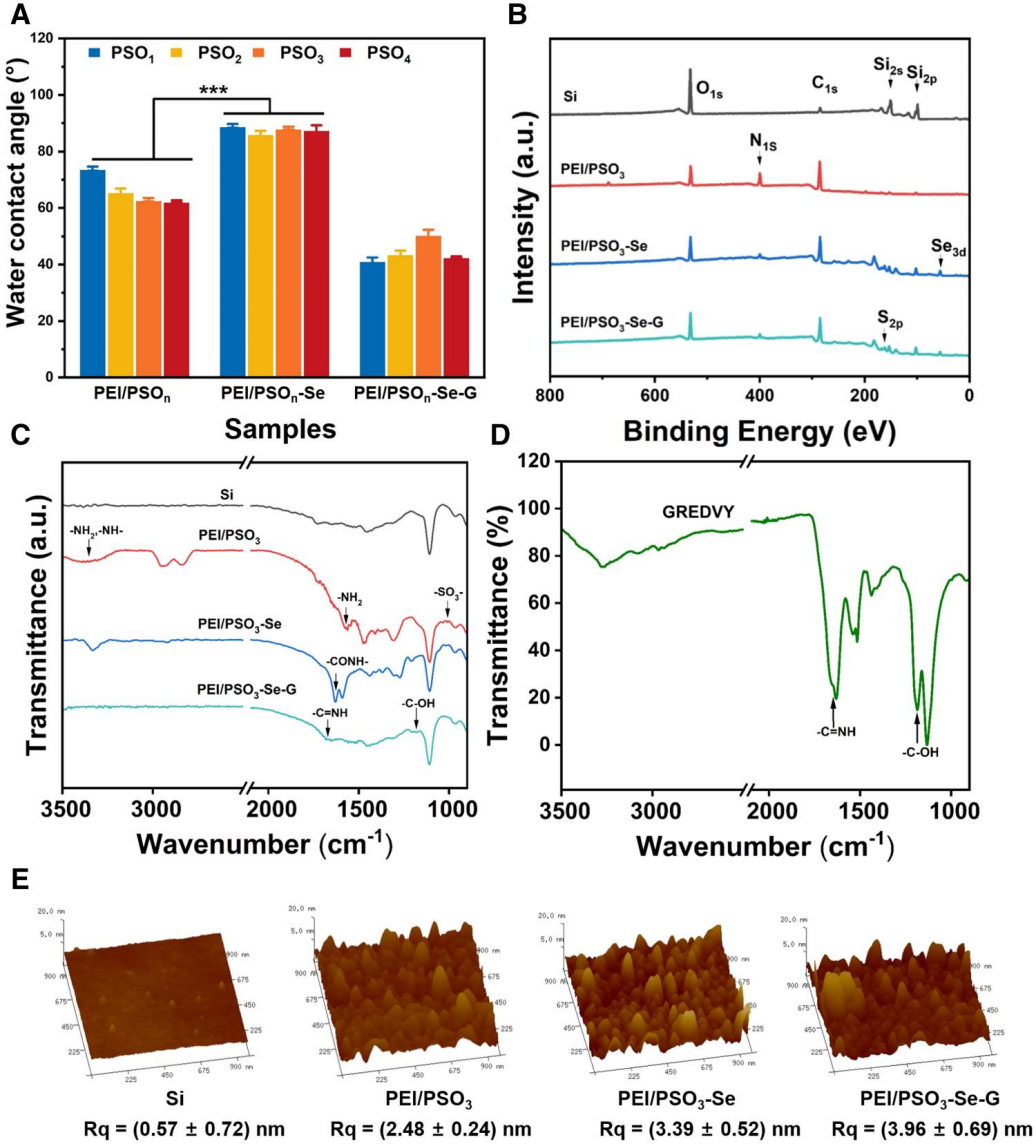
(**A**) Water contact angle, (**B**) XPS, (**C**), (**D**) FTIR and (**E**) AFM.

As shown in Figure 3A, the introduction of SeCA increased the water contact angle of the PEI/PSO_n_-Se surface from 70° to 90°. After incorporation of the GREDVY peptide, the water contact angle of the PEI/PSO_n_-Se-G surface decreased to 40°. As shown in Figure 3B, Se signal, which was not detected in the PEI/PSO_3_ samples, was observed on both the PEI/PSO_3_-Se and PEI/PSO_3_-Se-G surfaces. As shown in Figure 3C, a new absorption peak at 1627 cm^−1^, attributed to -CONH- [42], was observed for the PEI/PSO_3_-Se surface in the FTIR spectrum. The absorption bands at 1630 and 1190 cm^−1^ from the PEI/PSO_3_-Se-G surface was attributed to the -C=NH and -C-OH in GREDVY peptide (Figure 3D).

As shown in Figure 3E, the Si samples displayed a smooth surface, while granular structures with different sizes were observed on the modified surfaces. The root mean square roughness (Rq) of Si was (0.57 ± 0.72) nm. After the introduction of multilayers, Rq of the PEI/PSO_3_ surface was (2.48 ± 0.24) nm. The Rq values of the PEI/PSO_3_-Se surface and PEI/PSO_3_-Se-G surface were (3.39 ± 0.52) nm and (3.96 ± 0.69) nm, respectively. The data showed that the surface modification process resulted in slight changes in the surface morphology and roughness.

**Figure 4.**
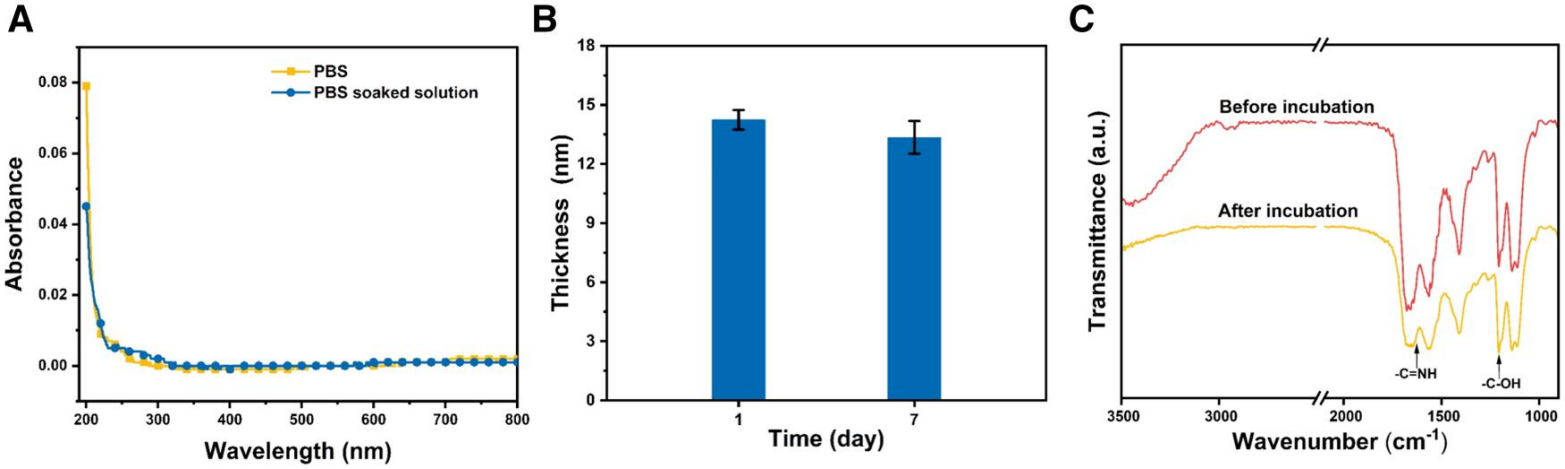
(**A**) Absorbance of the PBS solution before and after soaking the PEI/PSO_3_-Se-G samples for 7 days. Thicknesses (**B**) and FTIR spectra (**C**) of the PEI/PSO_3_-Se-G samples before and after 7 days of incubation in PBS solution.

The stability of the modified surfaces was investigated. Specifically, the PEI/PSO_3_-Se-G samples were incubated in PBS solution at 37°C for 7 days with shaking at 100 rpm. As shown in Figure 4A, the absorbance of the PBS solution before and after soaking the PEI/PSO_3_-Se-G samples was basically unchanged. The thicknesses of the PEI/PSO_3_-Se-G samples did not obviously differ before and after 7 days of incubation in PBS solution (Figure 4B). As shown in Figure 4C, after 7 days of incubation in PBS solution at 37°C, the absorption peaks at 1630 cm^−1^, attributed to -C=NH and 1190 cm^−1^, attributed to -C-OH were still present on the PEI/PSO_3_-Se-G surface, indicating that the peptide was still on the surface.

### *In vitro* catalytic generation of NO

**Figure 5.**
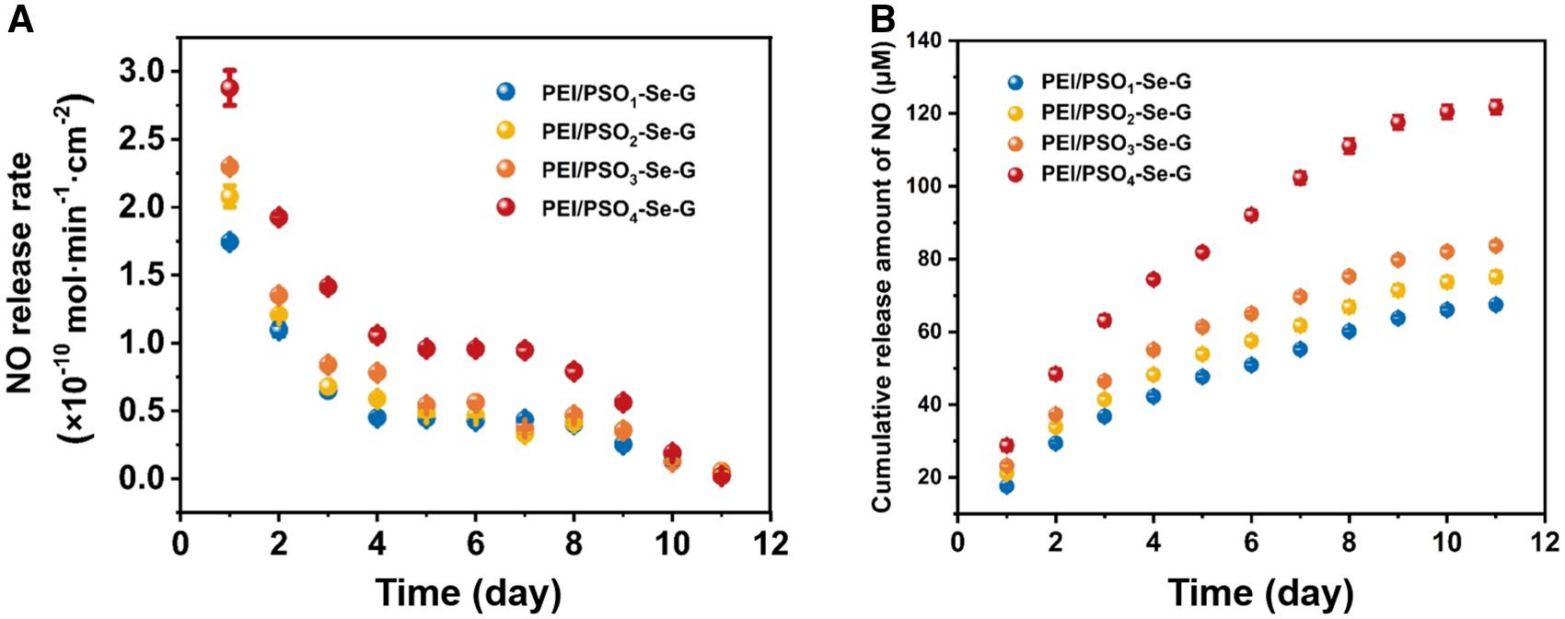
(**A**) NO Release rates of the PEI/PSO_n_-Se-G samples within a period of 11 days. NO release rate of Si was approximately 0.065 × 10^−10 ^mol·min^−1^·cm^−2^. (**B**) Cumulative release amount of NO catalyzed by different samples.

Selenocystamine was able to catalyze the production of NO from endogenous NO donors, as reported previously [43, 44]. The samples were placed in working solution, and the NO release rate was assessed using a Griess Assay Kit. As shown in Figure 5A, PEI/PSO_1_-Se-G, PEI/PSO_2_-Se-G, PEI/PSO_3_-Se-G and PEI/PSO_4_-Se-G maintained physiological levels of NO release rates ((0.54–2.87) ×10^−10 ^mol·min^−1^·cm^−2^) for 3, 4, 5 and 9 days, respectively. As shown in Figure 5B, it was concluded that NO release at multiple time points of different samples followed a logarithmic model; the kinetic equation can be described as *y* = *A* ln(*x*) + *B*. A similar phenomenon was observed in a previous report [45]. Supplementary Figure S5 shows the XPS survey spectra of different surfaces. Supplementary Table S4 shows elemental compositions measured by XPS. The ratios of Se/C on the PEI/PSO_1_-Se-G, PEI/PSO_2_-Se-G, PEI/PSO_3_-Se-G and PEI/PSO_4_-Se-G surfaces were 0.067, 0.070, 0.087 and 0.098, respectively. The gradual increase in atomic ratios of Se/C indicates a progressive increase in the Se content across the various surfaces. The higher the content of Se on the surface was, the greater the NO release rate and the longer the NO release longevity. The effect of the surface Se content on the rate of NO release has also been reported previously [46].

### Adhesion and proliferation of HUVSMCs

PEI/PSS-Se samples were able to maintain physiological levels of NO release rates ((0.56–1.48) ×10^−10 ^mol·min^−1^·cm^−2^) for 5 days (Supplementary Figure S6). However, many HUVSMCs adhered to the PEI/PSS-Se samples, similar to the number of HUVSMCs adhering to Si (Supplementary Figure S7). Therefore, a hydrophilic and antifouling component (POEGMA) was introduced to the surface to reduce cell adhesion.

#### 24 h of incubation

##### Without the donor

**Figure 6.**
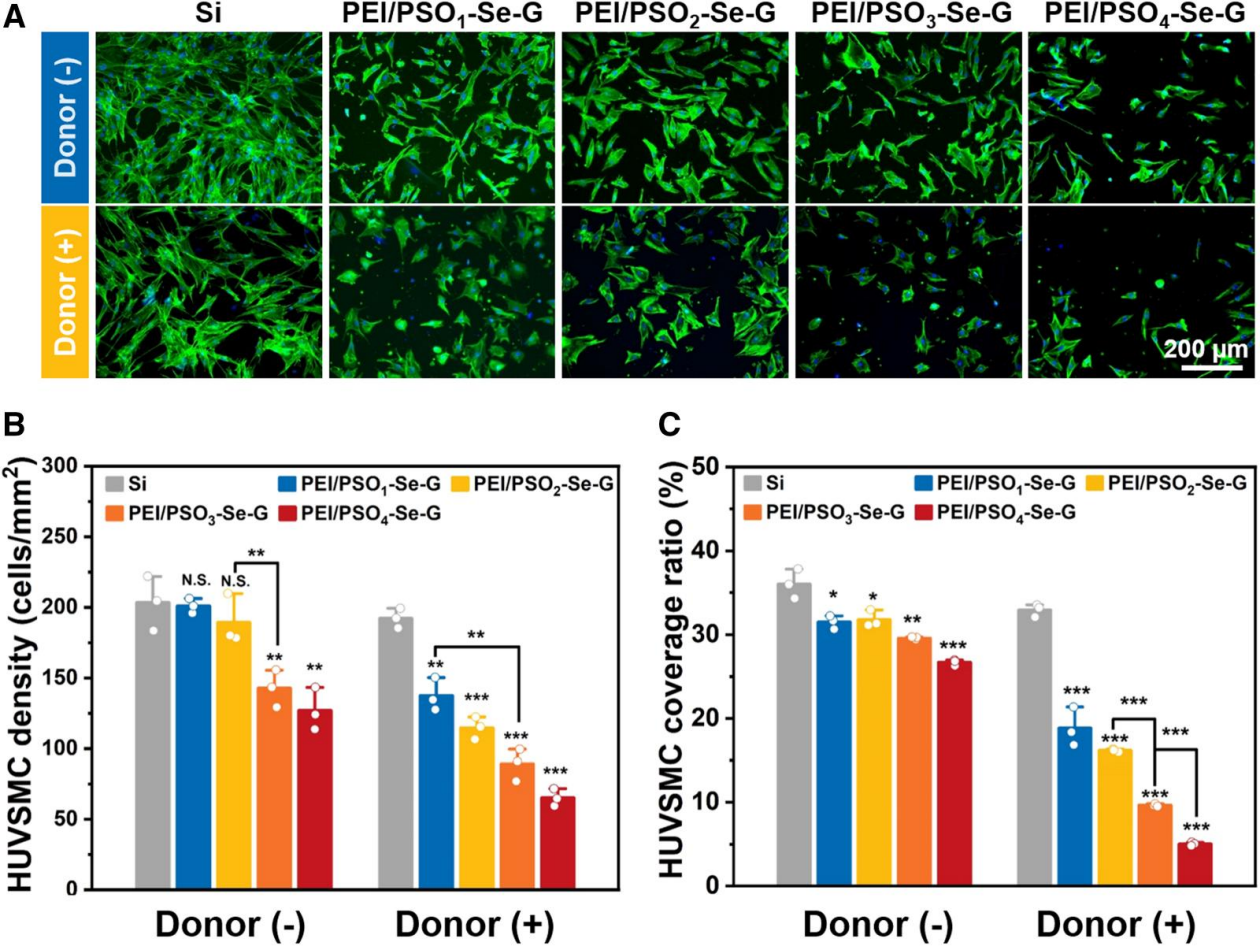
Fluorescence images (**A**), cell density (**B**) and cell coverage ratio (**C**) of HUVSMCs on different samples after 24 h of incubation.

After 24 h incubation without the donor, HUVSMCs cultured on different samples were spread with an irregular polygon morphology (Figure 6A). As shown in Figure 6B, the HUVSMC density on Si was approximately 203 cells/mm^2^. For the PEI/PSO_n_-Se-G samples, with increased *f*_POEGMA_ and decreased *f*_PSS_ in the PSO_n_ copolymers, the HUVSMC densities in the PEI/PSO_1_-Se-G, PEI/PSO_2_-Se-G, PEI/PSO_3_-Se-G and PEI/PSO_4_-Se-G samples gradually decreased to ∼201, ∼189, ∼142, and ∼127 cells/mm^2^, respectively. As shown in Supplementary Figure S8, HUVSMC viability on Si was ∼100%. The viabilities of HUVSMCs on PEI/PSO_1_-Se-G and PEI/PSO_2_-Se-G samples were >98%. As shown in Figure 6C, the cell coverage ratio on Si was ∼36%. The HUVSMC coverage ratios of the PEI/PSO_1_-Se-G, PEI/PSO_2_-Se-G, PEI/PSO_3_-Se-G and PEI/PSO_4_-Se-G samples were ∼31%, ∼31%, ∼29% and ∼26%, respectively, showing a decreasing trend. Compared with those on Si, the HUVSMC coverage ratios on the PEI/PSO_3_-Se-G and PEI/PSO_4_-Se-G samples were reduced by 60%. It was also observed in previous studies that combining antifouling component and cell promotion component was able to modulate cell adhesion [21, 29].

##### With the donor

After 24 h of incubation with the donor, the cell density and coverage ratio of HUVSMCs on Si were similar to those observed during incubation without the donor. However, HUVSMCs cultured on PEI/PSO_n_-Se-G samples were contractile with a punctate morphology after incubation with the donor as reported previously [47]. The HUVSMC densities on the PEI/PSO_1_-Se-G, PEI/PSO_2_-Se-G, PEI/PSO_3_-Se-G and PEI/PSO_4_-Se-G samples gradually decreased to ∼137, ∼114, ∼89, and ∼65 cells/mm^2^, respectively. The HUVSMC coverage ratios were ∼18%, ∼16%, ∼9% and ∼5%, respectively. Compared with those without the donor, the HUVSMC density and cell coverage ratio on PEI/PSO_n_-Se-G were significantly lower. In the presence of donor, PEI/PSO_n_-Se-G samples were able to decompose donor to produce NO, suggesting that NO inhibited HUVSMC adhesion as previously reported [7, 48].

#### 72 h of incubation

##### Without the donor

**Figure 7.**
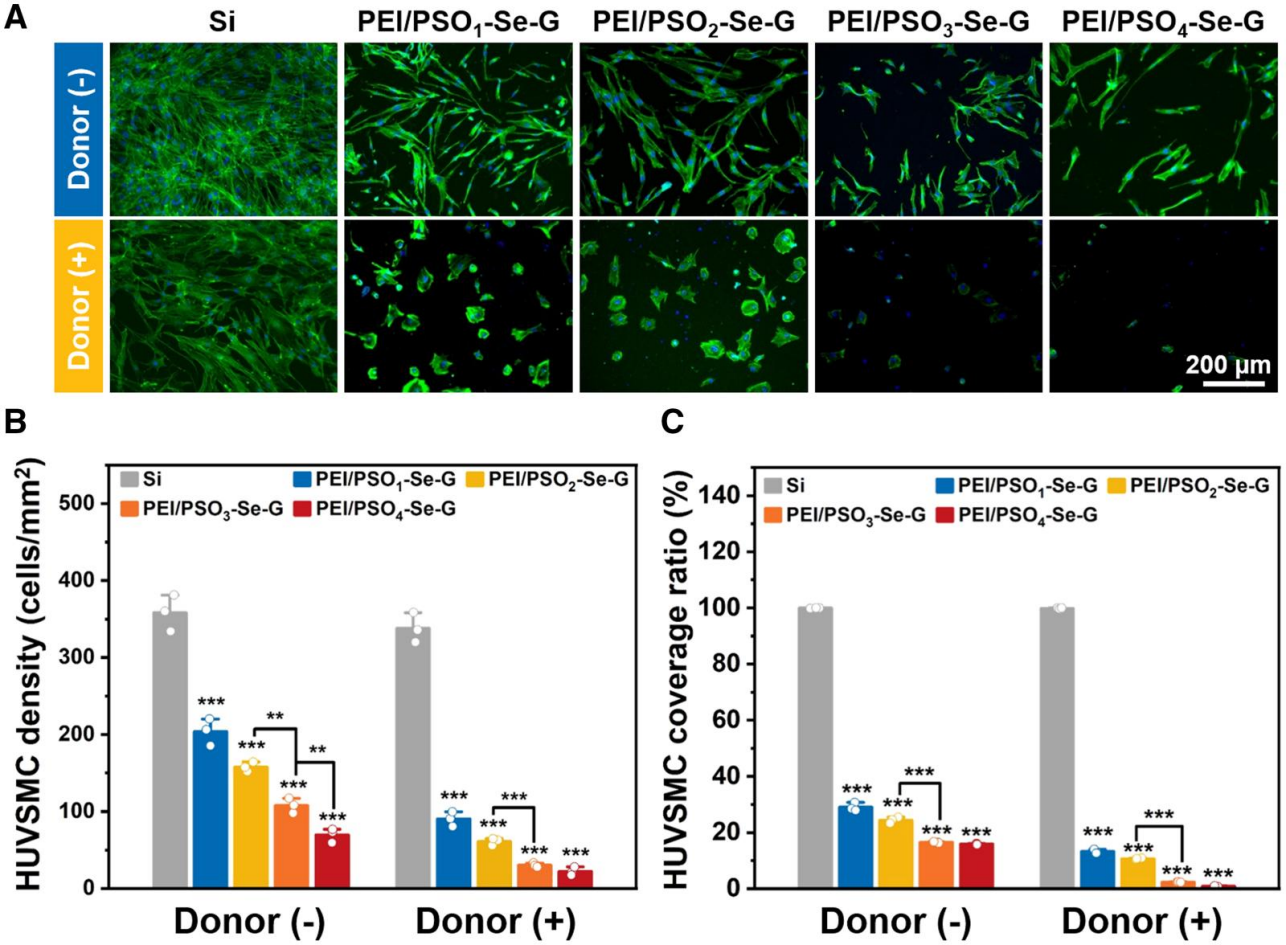
Fluorescence images (**A**), cell density (**B**) and cell coverage ratio (**C**) of HUVSMCs on different samples after 72 h of incubation.

After 72 h of incubation without the donor, HUVSMCs cultured on different samples were spread with an irregular polygon morphology (Figure 7A). As shown in Figure 7B, the HUVSMC density on Si was approximately 358 cells/mm^2^, and the HUVSMC densities on the PEI/PSO_1_-Se-G, PEI/PSO_2_-Se-G, PEI/PSO_3_-Se-G and PEI/PSO_4_-Se-G samples decreased gradually to ∼200, ∼157, ∼107 and ∼69 cells/mm^2^, respectively. As shown in Figure 7C, the cell coverage ratio on Si was ∼99%, and the HUVSMC coverage ratios of the PEI/PSO_1_-Se-G, PEI/PSO_2_-Se-G, PEI/PSO_3_-Se-G and PEI/PSO_4_-Se-G samples were approximately 29%, 24%, 16% and 15%, respectively, showing a decreasing trend. Compared with those on Si, the HUVSMC coverage ratios on the PEI/PSO_3_-Se-G and PEI/PSO_4_-Se-G samples were reduced by more than 84%.

##### With the donor

After 72 h of incubation with the donor, the HUVSMC density on Si was approximately 337 cells/mm^2^, and the cell coverage ratio on Si was ∼99%, with a spread morphology similar to that on Si during incubation without the donor. As shown in Figure 7A, HUVSMCs cultured on PEI/PSO_n_-Se-G samples maintained a contracted morphology. The HUVSMC densities on the PEI/PSO_1_-Se-G, PEI/PSO_2_-Se-G, PEI/PSO_3_-Se-G and PEI/PSO_4_-Se-G samples showed decreasing trends of approximately 90, 61, 30, and 22 cells/mm^2^, respectively. The HUVSMC coverage ratios of the PEI/PSO_1_-Se-G, PEI/PSO_2_-Se-G, PEI/PSO_3_-Se-G and PEI/PSO_4_-Se-G samples gradually decreased to ∼13%, ∼10%, ∼2% and ∼1%, respectively. Compared with those on Si, the HUVSMC coverage ratios on the PEI/PSO_3_-Se-G and PEI/PSO_4_-Se-G samples during incubation with the donor decreased by more than 97%.

For the PEI/PSO_n_-Se-G samples, with increased *f*_POEGMA_ and decreased *f*_PSS_ in the PSO_n_ copolymers, whatever without the donor or with the donor, the cell density and coverage ratio of HUVSMCs decreased gradually. The addition of donors further decreased the HUVSMC density and cell coverage ratio, and it was speculated that NO catalytically generated by the PEI/PSO_n_-Se-G samples during incubation with the donor inhibited the adhesion and proliferation of HUVSMCs [23, 49]. The results showed that NO and chemical composition acted synergistically to inhibit HUVSMCs.

### The expression level of cGMP in HUVSMCs

During incubation with the donor, the proliferation of HUVSMCs cultured on PEI/PSO_n_-Se-G samples was inhibited significantly. This was especially true for the PEI/PSO_3_-Se-G and PEI/PSO_4_-Se-G samples. It was reported that NO inhibited the growth of smooth muscle cells by promoting cGMP levels in the vascular system [46, 50, 51]. Herein, HUVSMCs were seeded on the PEI/PSO_3_-Se-G samples to investigate the effect of NO on cGMP expression in HUVSMCs.

**Figure 8.**
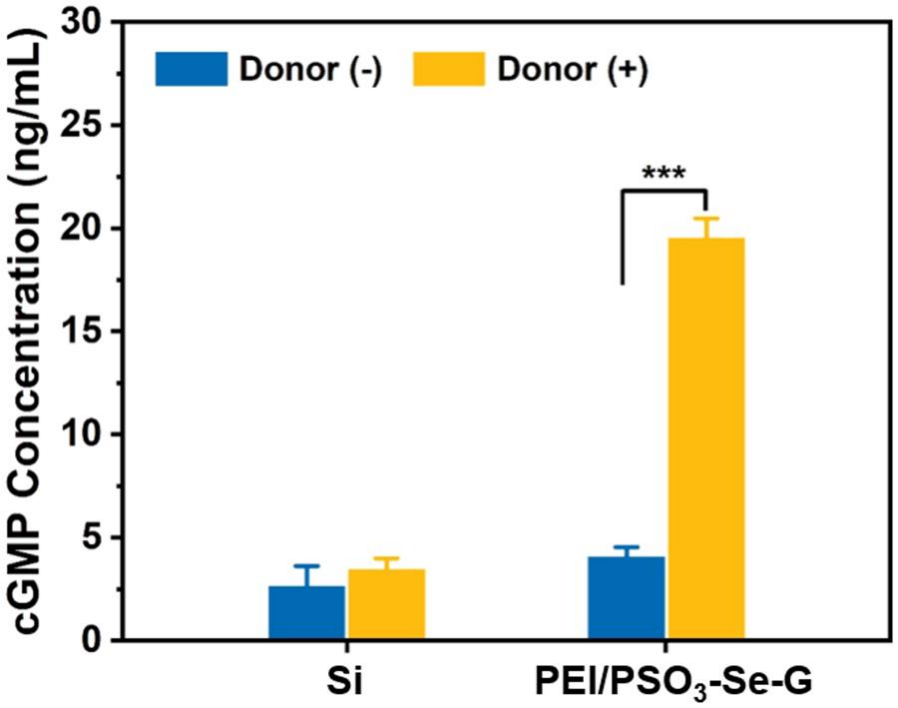
cGMP concentrations in HUVSMCs cultured on Si and PEI/PSO_3_-Se-G samples for 2 h.

The cGMP concentrations in HUVSMCs after 2 h of incubation are shown in Figure 8. For HUVSMCs cultured on Si, the cGMP concentration was ∼2.7 ng/ml during incubation without the donor and ∼3.5 ng/ml during incubation with the donor. The addition of donors did not significantly affect the cGMP concentration in HUVSMCs cultured on Si.

For HUVSMCs cultured on PEI/PSO_3_-Se-G samples for 2 h without the donor, the cGMP concentration was ∼4.1 ng/ml. In contrast, the cGMP concentration in HUVSMCs cultured on PEI/PSO_3_-Se-G samples with the donor was ∼20 ng/ml, which was 4.9-fold greater than that observed during incubation without the donor. The addition of donors significantly increased the cGMP concentration in HUVSMCs as previously reported [51, 52]. These data suggested that the presence of NO catalyzed by PEI/PSO_3_-Se-G during incubation with the donor improved the cGMP level to regulate the adhesion and proliferative behavior of HUVSMCs [53].

### The expression level of α-SMA in HUVSMCs

**Figure 9.**
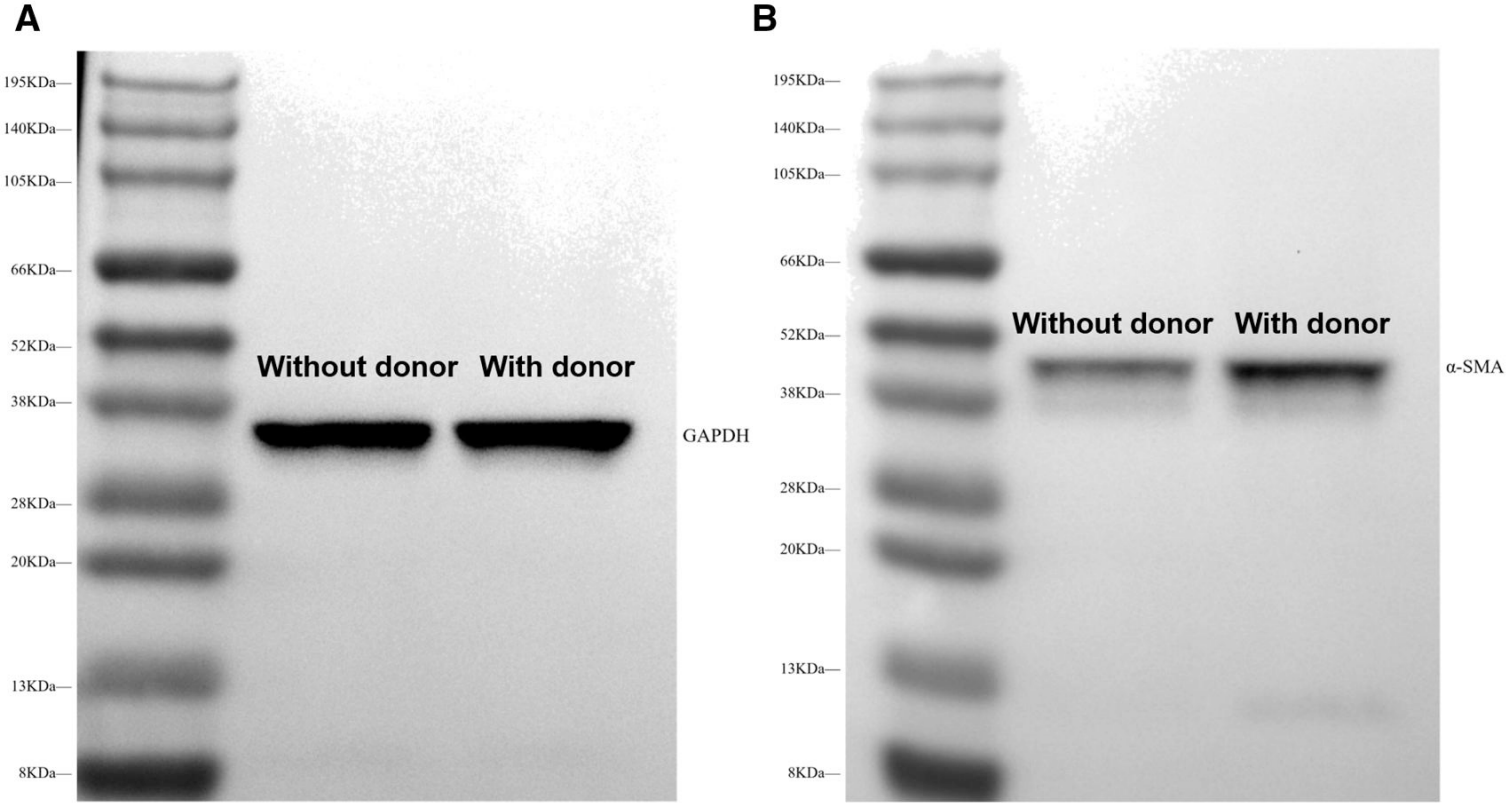
Western blotting analysis of α-SMA in the HUVSMCs on PEI/PSO_3_-Se-G samples after 72 h of incubation. (**A**) GAPDH and (**B**) α-SMA.

In the NO-cGMP pathway, α-SMA is a key signaling molecule for controlling HUVSMCs with a typical contractile phenotype [54]. After 72 h of incubation of HUVSMCs with PEI/PSO_3_-Se-G samples, the expression of α-SMA in HUVSMCs was evaluated by Western blotting. As shown in Figure 9A, GAPDH was used as a reference protein. The addition of donors did not significantly affect the expression level of GAPDH in HUVSMCs. Compared with HUVSMCs cultured without donors, HUVSMCs cultured with donors exhibited upregulated α-SMA expression (Figure 9B). The PEI/PSO_3_-Se-G samples specifically suppressed the overgrowth of HUVSMCs, and maintained their contractile phenotype by upregulating the expression of α-SMA in the NO-cGMP pathway [10, 19].

### Adhesion and proliferation of HUVECs

#### 24 h of incubation

##### Without the donor

**Figure 10.**
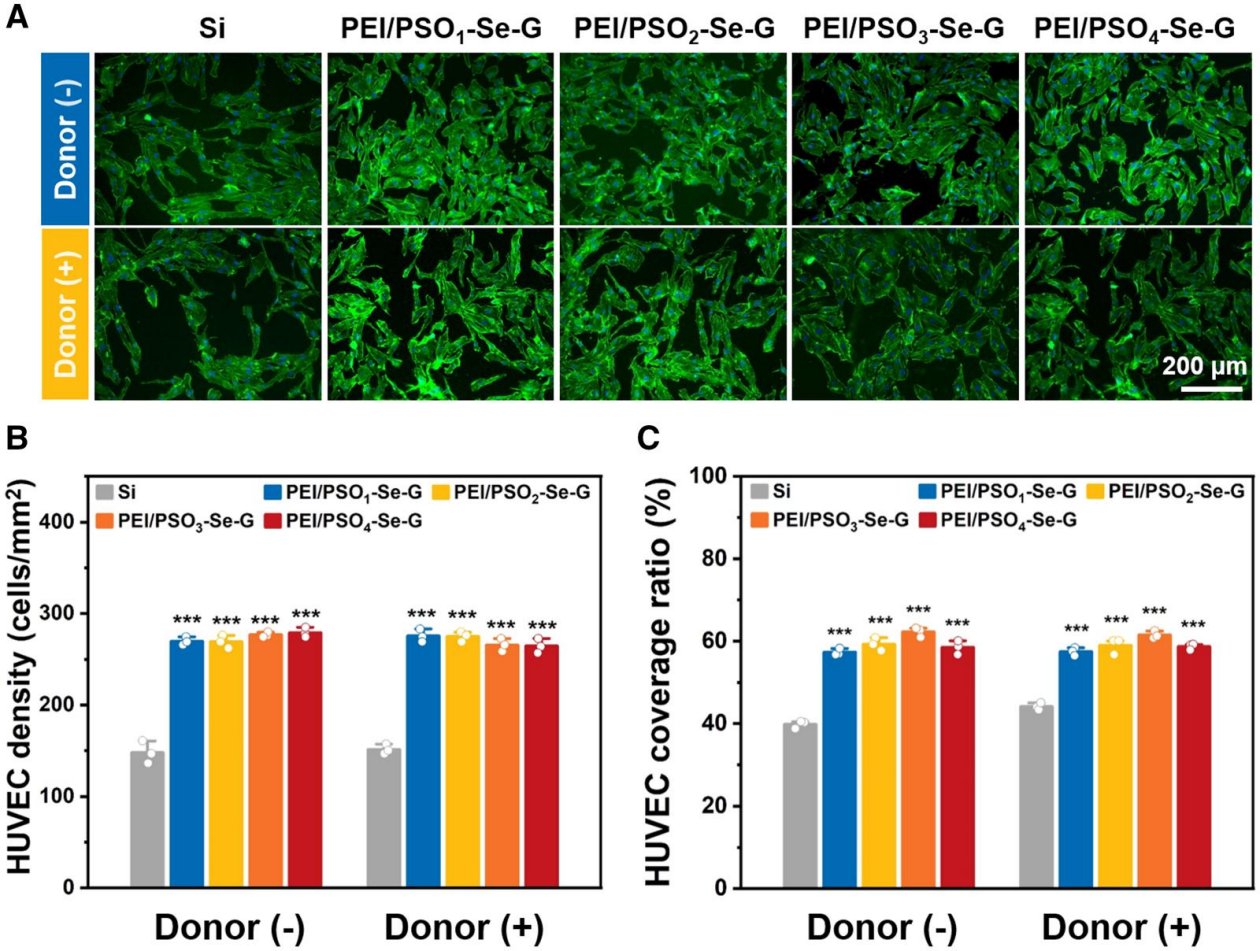
Fluorescence images (**A**), cell density (**B**) and cell coverage ratio (**C**) of HUVECs on different samples after 24 h of incubation.

After 24 h of incubation without the donor, HUVECs on different samples were spread excellently with a polygonal morphology, as shown in Figure 10A. The HUVEC density on Si was approximately 150 cells/mm^2^ and the HUVEC coverage ratio on Si was ∼40%. The HUVEC density on the PEI/PSO_n_-Se-G samples was approximately 280 cells/mm^2^, which was 1.9-fold greater than that on the Si sample (Figure 10B). The HUVEC coverage ratio on PEI/PSO_n_-Se-G samples was approximately 60%, which was 1.5-fold greater than that of the Si sample (Figure 10C). HUVEC viability on Si was ∼80%, while HUVEC viability on PEI/PSO_n_-Se-G samples was ∼100% (Supplementary Figure S9C). The results suggested that the PEI/PSO_n_-Se-G samples significantly enhanced the adhesion of HUVECs.

##### With the donor

After 24 h of incubation with the donor, the HUVEC morphology, cell density, coverage ratio and cell viability of the PEI/PSO_n_-Se-G samples were similar to those of the samples without the donor. The presence of NO had no significant effect on HUVECs after 24 h of incubation.

#### 72 h of incubation

**Figure 11.**
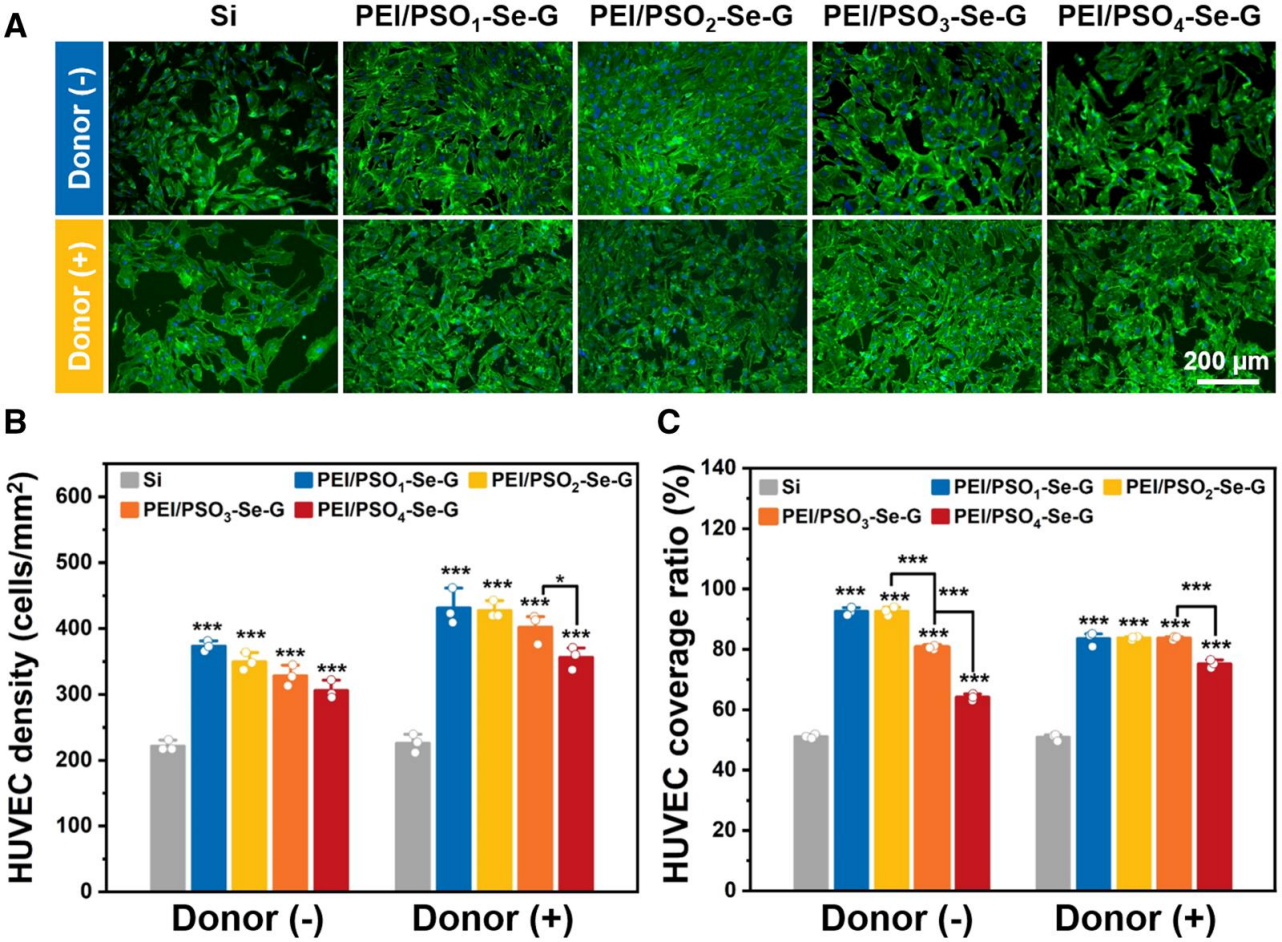
Fluorescence images (**A**), cell density (**B**) and cell coverage ratio (**C**) of HUVECs on different samples after 72 h of incubation.

HUVECs cultured on different samples for 72 h are shown in Figure 11. Regardless of whether there was a donor, the HUVEC viability on Si was ∼80%, and the HUVEC viability on PEI/PSO_n_-Se-G samples was ∼100% (Supplementary Figure S9D). HUVECs cultured on different samples spread heavily (Figure 11A), and the cell morphology was similar to that after 24 h of incubation. The HUVEC density and coverage ratio on the Si and PEI/PSO_n_-Se-G samples increased significantly compared with those after 24 h of incubation.

##### Without the donor

After 72 h of incubation without the donor, the HUVEC density on Si was approximately 220 cells/mm^2^, and the coverage ratio was ∼50%. The HUVEC densities on the PEI/PSO_1_-Se-G, PEI/PSO_2_-Se-G, PEI/PSO_3_-Se-G and PEI/PSO_4_-Se-G samples were ∼372, ∼349, ∼328 and ∼305 cells/mm^2^, respectively (Figure 11B). The HUVEC coverage ratios of PEI/PSO_1_-Se-G, PEI/PSO_2_-Se-G, PEI/PSO_3_-Se-G and PEI/PSO_4_-Se-G were ∼92%, ∼92%, ∼80% and ∼64%, respectively, showing a decreasing trend (Figure 11C). As reported previously, a hydrophilic component was able to inhibit cell adhesion [5, 29] while a heparin analog promoted cell adhesion [34, 35]. It was hypothesized that combining the antifouling component and cell promotion component could regulate the HUVEC density and cell coverage ratio on the PEI/PSO_n_-Se-G samples.

##### With the donor

After 72 h of incubation with the donor, the HUVEC density and cell coverage ratio on Si were similar to those without the donor. The HUVEC densities on the PEI/PSO_1_-Se-G, PEI/PSO_2_-Se-G, PEI/PSO_3_-Se-G and PEI/PSO_4_-Se-G samples were ∼431, ∼427, ∼402 and ∼356 cells/mm^2^, respectively. The HUVEC density on the PEI/PSO_n_-Se-G samples increased when the donor was added. In particular, compared with that on the non-donor sample, the density of HUVECs on the PEI/PSO_3_-Se-G sample during incubation with the donor had the most significant increase (∼33%). The HUVECs cultured on the PEI/PSO_n_-Se-G samples were all spread out, and the coverage ratios of PEI/PSO_1_-Se-G, PEI/PSO_2_-Se-G, PEI/PSO_3_-Se-G and PEI/PSO_4_-Se-G were ∼83%, ∼80%, ∼83%, and ∼75%, respectively (Figure 11C).

After 24 h of incubation, for HUVECs cultured on PEI/PSO_n_-Se-G samples, the chemical composition of the PSO_n_ copolymer and the addition of the donor had no significant effect on the HUVEC density or coverage ratio. In contrast, after 72 h of incubation, the addition of a donor increased the HUVEC density on the PEI/PSO_n_-Se-G samples. In the long run, NO catalytically generated by the PEI/PSO_n_-Se-G samples when the donor was added promoted HUVECs as reported previously [55, 56].

It is noted that the PEI/PSO_n_-Se surface did not significantly promote HUVEC proliferation when the donor was added (Supplementary Figure S10). After the incorporation of the GREDVY peptide on the surface, the PEI/PSO_n_-Se-G samples became more friendly to the HUVECs. The ability of the GREDVY peptide to promote HUVEC proliferation was also observed in previous studies [57, 58].

### The expression level of CD31 in HUVECs

**Figure 12.**
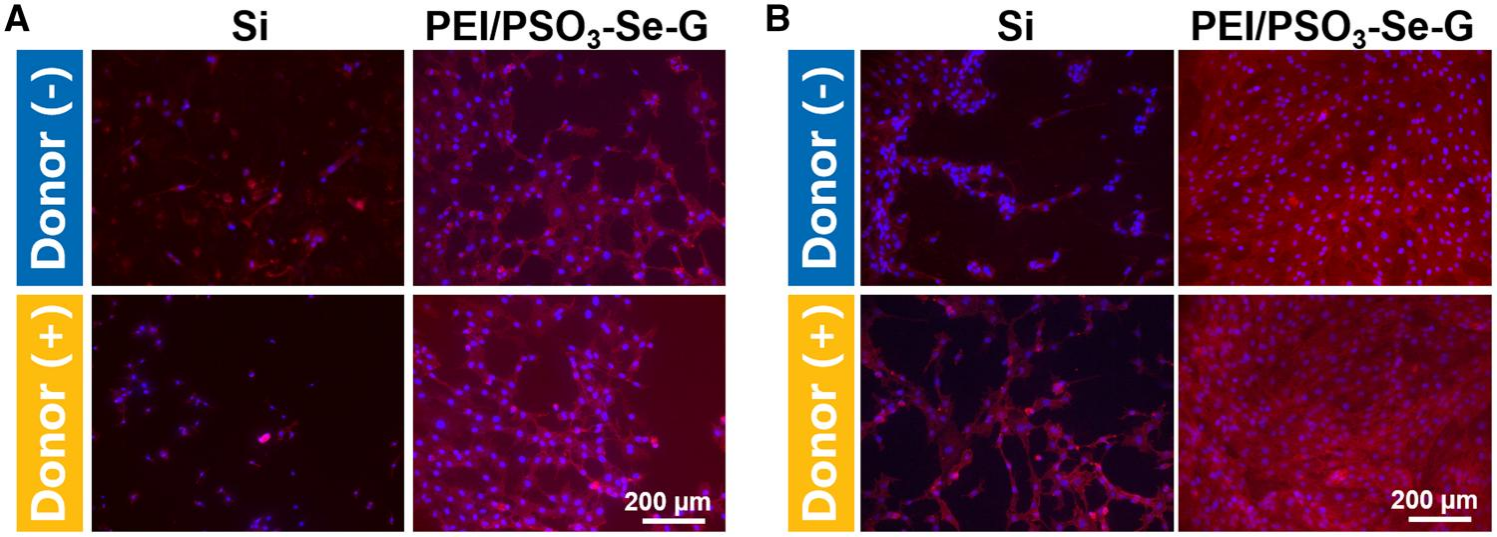
Immunofluorescence images of HUVECs on different samples after 24 h (**A**) and 72 h (**B**) of incubation.

Immunofluorescence staining was performed to detect the expression of an endothelial specific marker (CD31) in HUVECs [54, 59]. After 24 h of incubation, the immunofluorescence intensity of CD31 in HUVECs cultured on the PEI/PSO_3_-Se-G sample was stronger compared to that on the Si control sample (Figure 12A). The addition of donors did not significantly affect the expression level of CD31 in HUVECs.

HUVECs cultured on different samples for 72 h are shown in Figure 12B. The CD31 immunofluorescence intensity of HUVECs on the Si and PEI/PSO_3_-Se-G samples increased significantly compared with that after 24 h of incubation. After 72 h of incubation without the donor, HUVECs spread to form a monolayer with strong immunofluorescence intensity of CD31 on the PEI/PSO_3_-Se-G samples. The addition of donors did not significantly affect the expression level of CD31 in HUVECs.

The data suggested that compared with the Si sample, the PEI/PSO_3_-Se-G sample significantly promoted HUVECs proliferation. This trend is in accordance with the results for the adhesion and proliferation of HUVECs.

### Migration of HUVECs

The migratory capacity of HUVECs plays an important role in the recovery of damaged endothelial layers and endothelialization [17, 60, 61]. Therefore, the migration behavior of HUVECs on PEI/PSO_n_-Se-G surfaces was investigated.

#### Without the donor

**Figure 13.**
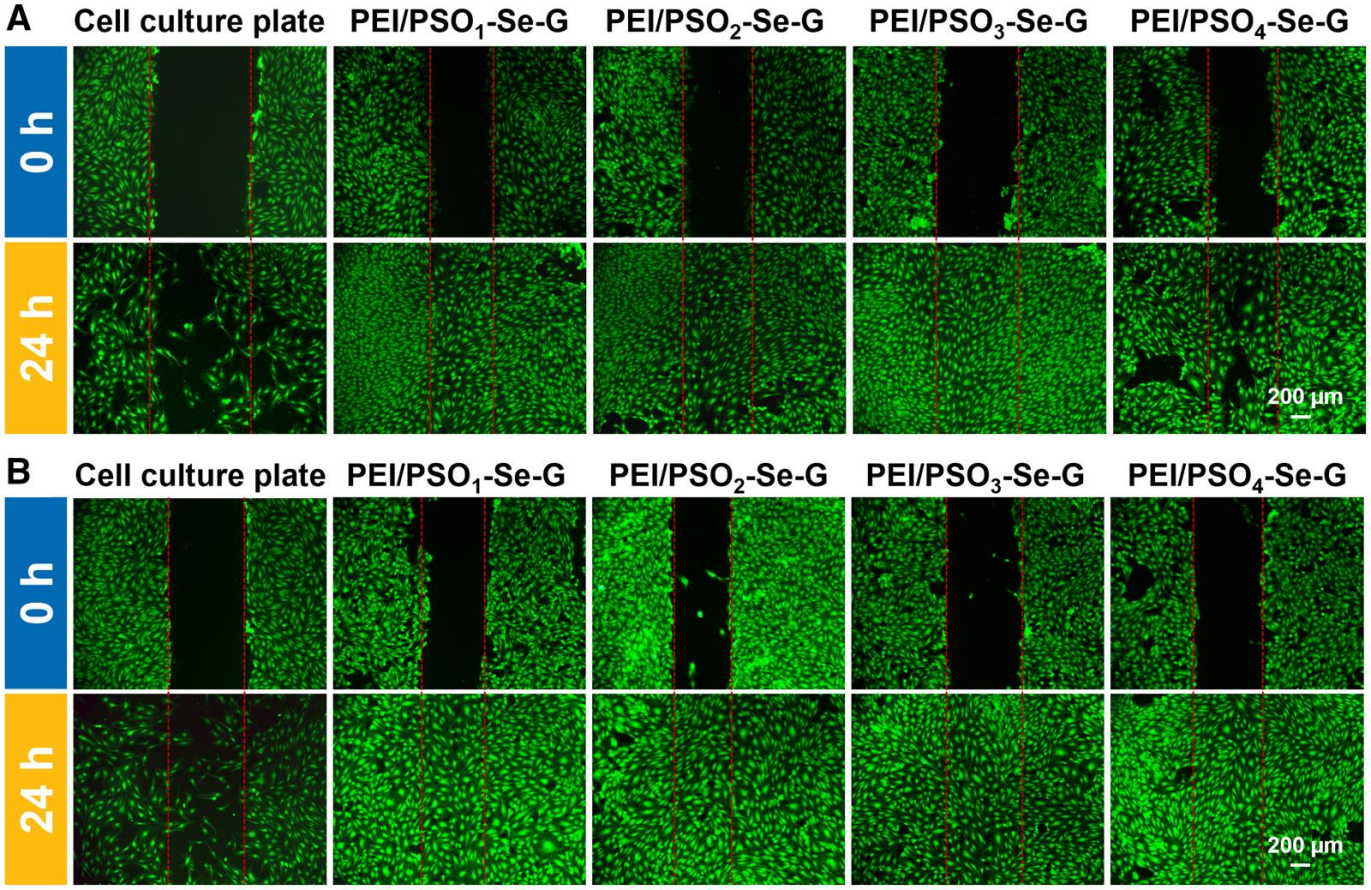
Fluorescence images showing the migration of HUVECs cultured on different samples after scratching with a pipette tip. Migration of HUVECs after 24 h of incubation without the donor (**A**) and with the donor (**B**).

As shown in Figure 13, 0 and 24 h of incubation represented the state of HUVECs when the scratches were created and the state of HUVECs continuing to incubate for 24 h after making scratches, respectively. After 24 h of incubation without the donor, HUVECs cultured on the cell culture plate migrated to cover approximately 18% of the scratched area. However, HUVECs cultured on the PEI/PSO_1_-Se-G, PEI/PSO_2_-Se-G, PEI/PSO_3_-Se-G samples migrated significantly, covering approximately 100% of the scratched area, except for those cultured on the PEI/PSO_4_-Se-G samples, which had 76% migration coverage (Figure 13A). The data suggested that the high content of the POEGMA component in the PSO_4_ copolymers inhibited the adhesion and migration of HUVECs cultured on the PEI/PSO_4_-Se-G samples [15].

#### With the donor

After 24 h of incubation with the donor (Figure 13B), HUVECs cultured on the cell culture plate migrated to cover approximately 27% of the scratched area. However, the scratched areas on the PEI/PSO_n_-Se-G samples were all significantly reduced and almost completely healed. Even HUVECs cultured on PEI/PSO_4_-Se-G samples were overgrown. The presence of NO catalytically generated by PEI/PSO_n_-Se-G samples promoted the migration of HUVECs. Similar results were also observed in previous studies [10, 62]. The migration of HUVECs showed that multifunctional surfaces that included four components were able to significantly promote endothelialization.

### Co-culture of HUVECs and HUVSMCs

When blood vessels are damaged, HUVECs and HUVSMCs compete with each other. Compared with HUVECs, HUVSMCs have a greater proliferative capacity. Overgrowth of HUVSMCs causes problems such as endothelial hyperplasia [2, 63]. Therefore, evaluating the competitive growth of HUVECs relative to that of HUVSMCs on modified surfaces is essential.

**Figure 14.**
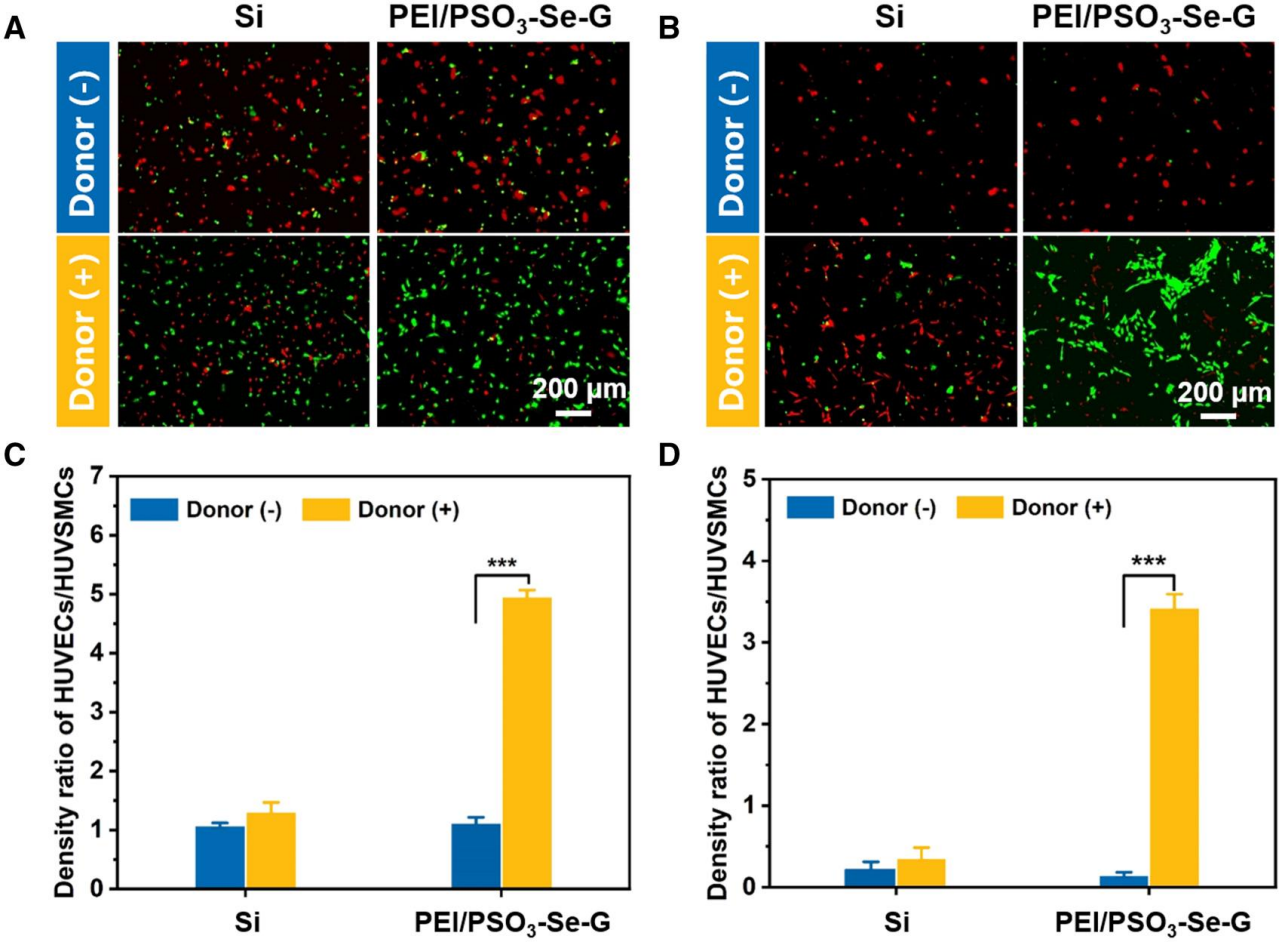
Competitive growth behaviors of HUVECs and HUVSMCs on Si and PEI/PSO_3_-Se-G samples. Fluorescence images after co-culture for 2 h (**A**) and 24 h (**B**). density ratios of HUVECs/HUVSMCs after co-culture for 2 h (**C**) and 24 h (**D**).

Based on the results of separate cultures of HUVECs and HUVSMCs, PEI/PSO_3_-Se-G samples were used to observe the co-culture conditions. HUVECs and HUVSMCs were labeled with green cell-tracker and red cell-tracker, respectively. Fluorescence images and density ratios of HUVECs/HUVSMCs after 2 and 24 h of incubation are shown in Figure 14.

#### 2 h of incubation

After 2 h of incubation without the donor, HUVECs and HUVSMCs were distributed uniformly with a dot shape on the Si and PEI/PSO_3_-Se-G samples (Figure 14A). The density ratios of HUVECs/HUVSMCs on the Si and PEI/PSO_3_-Se-G samples were approximately 1 (Figure 14C), with no significant difference between the Si and PEI/PSO_3_-Se-G samples. After 2 h of incubation with the donor, HUVECs and HUVSMCs adhered uniformly to the Si. The density ratio of HUVECs/HUVSMCs on Si was ∼1, similar to the ratio observed during incubation without the donor. However, more HUVECs than HUVSMCs adhered to the PEI/PSO_3_-Se-G samples (Figure 14A), and the density ratio of HUVECs/HUVSMCs on the PEI/PSO_3_-Se-G samples was ∼4.9 (Figure 14C).

#### 24 h of incubation

After 24 h of incubation without the donor, fewer HUVECs adhered to the Si and PEI/PSO_3_-Se-G samples (Figure 14B) than after 2 h of incubation without the donor. The density ratios of HUVECs/HUVSMCs on the Si and PEI/PSO_3_-Se-G samples were approximately 0.2 (Figure 14D). After 24 h of incubation with the donor, the HUVSMCs heavily adhered to the Si, and the density ratio of HUVECs/HUVSMCs on the Si was ∼0.3. However, much fewer HUVSMCs were spread on the PEI/PSO_3_-Se-G samples (Figure 14B). The number of HUVSMCs on PEI/PSO_3_-Se-G decreased significantly compared with that during incubation without the donor. The density ratio of HUVECs/HUVSMCs on PEI/PSO_3_-Se-G samples during incubation with the donor was ∼3.5 (Figure 14D).

These data showed that PEI/PSO_3_-Se-G samples enhanced the competitiveness of HUVECs over HUVSMCs in the presence of NO during incubation with the donor [64].

## Conclusions

In this work, a multifunctional surface for achieving endothelialization was constructed by LBL self-assembly with great simplicity. By adjusting the chemical composition of poly(sodium *p*-styrenesulfonate-*co*-oligo(ethylene glycol) methacrylate), the functions of the four components, heparin analogs, hydrophilic polymers, organic selenium and cell-adhesive peptide were balanced on the surface to achieve endothelialization. NO catalytically generated by PEI/PSO_n_-Se-G samples during incubation with the donor significantly inhibited HUVSMCs, and the incorporation of Gly-Arg-Glu-Asp-Val-Tyr peptide promoted HUVEC proliferation significantly. In particular, the PEI/PSO_3_-Se-G samples effectively inhibited HUVSMCs by upregulating the expression level of cGMP and α-SMA in HUVSMCs, and promoted HUVECs with the strong expression of CD31. The density ratio of HUVECs/HUVSMCs on the PEI/PSO_3_-Se-G samples was approximately 4.9 in the co-culture system. This strategy for achieving surface endothelialization may provide guidance for surface modification of biomaterials and biomedical devices.

## Supplementary Material

rbae096_Supplementary_Data
