# Supplementary material for: Balancing functions of antifouling, nitric oxide release and vascular cell selectivity for enhanced endothelialization of assembled multilayers
Source: Regen Biomater. 2024 Aug 24;11:rbae096. doi: 10.1093/rb/rbae096 (PMC11422184; doi:10.1093/rb/rbae096)
Supplement: rbae096_Supplementary_Data [file rbae096_supplementary_data.zip › Supplementary material.docx]

**Supporting Material**

Balancing functions of antifouling, nitric oxide release and vascular cell selectivity for enhanced endothelialization of assembled multilayers

Sulei Zhang ^a^, Jun Sun ^a^, Shuaihang Guo ^a^, Yichen Wang ^a^, Yuheng Zhang ^a^,

Jiao Lei ^a^, Xiaoli Liu ^a,^ *, Hong Chen ^a, b,^ *

^a^ *State and Local Joint Engineering Laboratory for Novel Functional Polymeric Materials, College of Chemistry, Chemical Engineering and Materials Science, Soochow University, Suzhou 215123, P. R. China*

^b^ *The SIP Biointerface Engineering Research Institute, Suzhou 215123, P. R. China*

**Corresponding Authors**

Email: liuxiaoli@suda.edu.cn (X. Liu),

chenh@suda.edu.cn (H. Chen)

**Table of Contents**

M1. GPC spectra of different copolymers

M2. NO release and HUVEC density of samples with different bilayers

M3. Chemical compositions of surfaces before and after piranha treatment

M4. Chemical compositions for each layer coating

M5. Zeta potential of modified SiO_2_ nanoparticles after each layer coating

M6. QCM-D measurement for quantifying polymers and peptide

M7. XPS survey spectra and chemical compositions of different samples

M8. NO release of PEI/PSS-Se samples

M9. HUVSMCs cultured on PEI/PSS-Se

M10. Cell viability of HUVECs and HUVSMCs

M11. HUVECs cultured on PEI/PSO_n_-Se

**M1. GPC spectra of different copolymers**

**
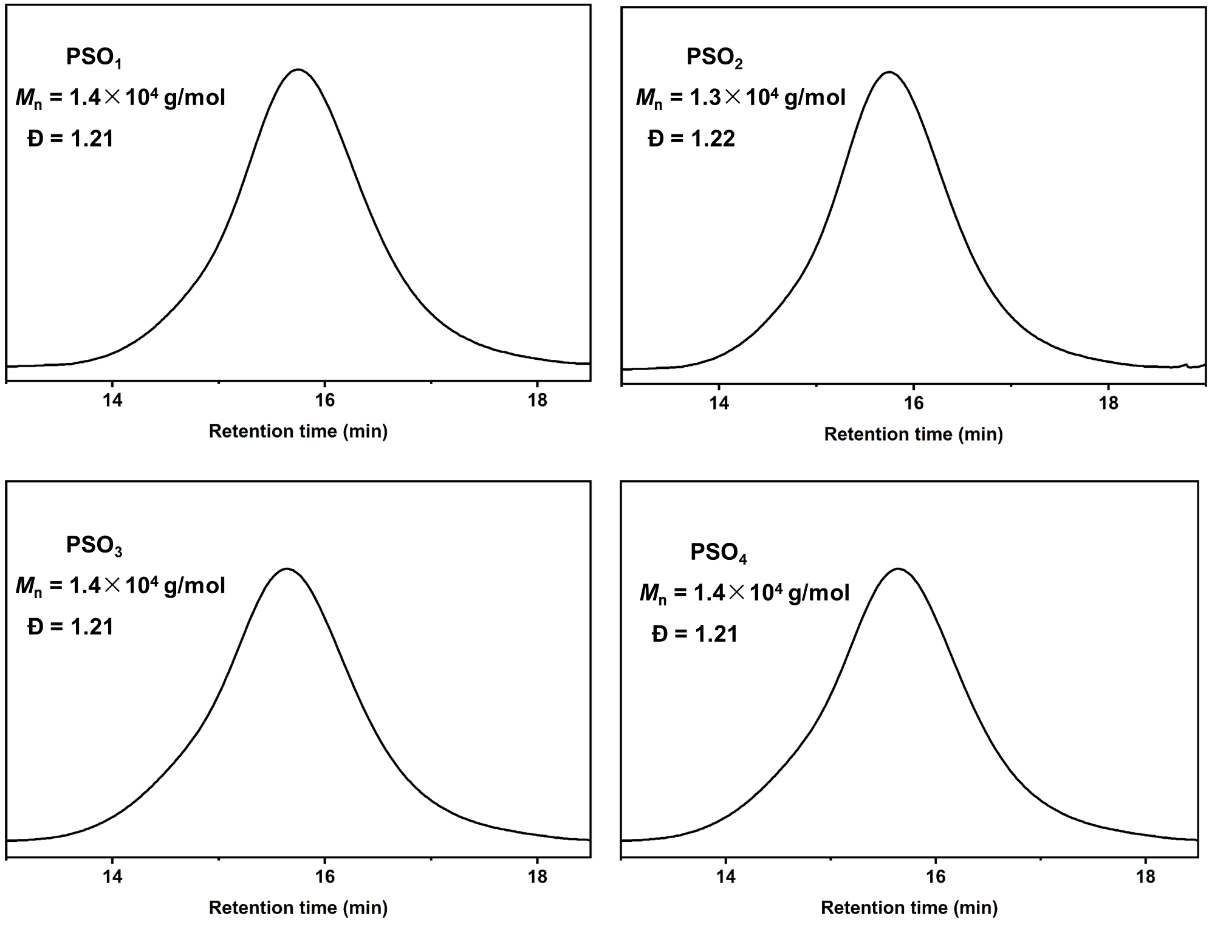
**

**Figure S1**. The GPC spectra of different copolymers.

**M2. NO release and HUVEC density of samples with different bilayers**

**
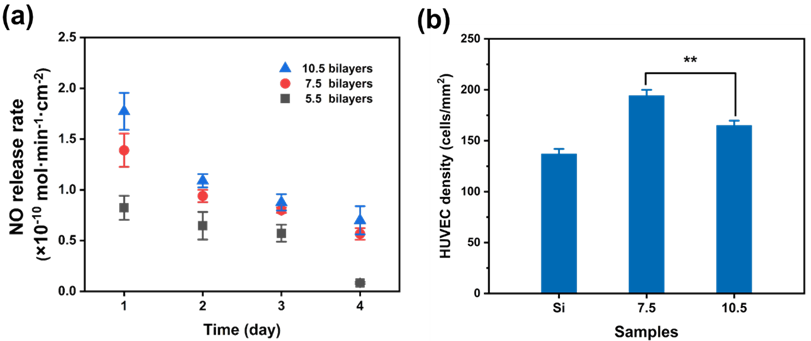
**

**Figure S2**. (a) NO release rates of the PEI/PSS-Se samples with different bilayers within 4 days. The NO release rate of Si sample was approximately 0.065 ×10^-10^ mol∙min^-1^∙cm^-2^. (b) HUVEC density on the PEI/PSS-Se samples with different bilayers after 48 h of culture with the donor (n = 3, ***P* < 0.01).

**M3. Chemical compositions of surfaces before and after piranha treatment**

**Table S1** The chemical compositions of Si and Si-OH surfaces detected by XPS

| **Samples** | **Elemental composition (%)** | | | O/Si |
| --- | --- | --- | --- | --- |
|  | C | Si | O |  |
| Si | 16.46 | 50.08 | 33.46 | 0.66 |
| Si-OH | 63.79 | 1.33 | 14.02 | 10.54 |

**M4. Chemical compositions for each layer coating**

**Table S2** The chemical compositions of different samples detected by XPS

(N.D. for non-detectable)

| **Bilayers** | **Elemental composition (%)** | | | | |
| --- | --- | --- | --- | --- | --- |
|  | C | Si | O | N | S |
| 0.5 | 65.30 | 0.90 | 10.13 | 23.67 | N.D. |
| 1 | 63.52 | 1.13 | 22.37 | 7.44 | 5.54 |
| 1.5 | 53.04 | 9.09 | 22.20 | 15.67 | N.D. |
| 2 | 59.88 | 5.09 | 22.41 | 7.01 | 5.61 |
| 2.5 | 61.14 | 3.88 | 10.70 | 23.08 | 1.20 |
| 3 | 63.52 | 1.52 | 22.10 | 7.27 | 5.59 |
| 3.5 | 68.93 | 1.28 | 4.93 | 24.38 | 0.47 |
| 4 | 64.12 | 0.73 | 21.20 | 8.06 | 5.90 |
| 4.5 | 62.79 | 3.15 | 10.11 | 23.00 | 0.96 |
| 5 | 64.20 | N.D. | 22.14 | 7.91 | 5.74 |
| 5.5 | 61.12 | 4.53 | 12.45 | 20.95 | 0.94 |
| 6 | 64.47 | 0.68 | 21.95 | 7.12 | 5.79 |
| 6.5 | 65.14 | 1.89 | 17.26 | 15.72 | N.D. |
| 7 | 64.40 | N.D. | 22.24 | 7.38 | 5.98 |
| 7.5 | 61.12 | 4.53 | 12.45 | 20.96 | 0.94 |

**M5. Zeta potential of modified SiO_2_ nanoparticles after each layer coating**


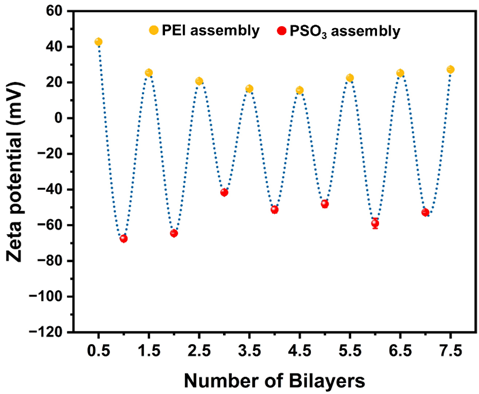


**Figure S3**. The zeta potential of the modified SiO_2_ nanoparticles in ultrapure water measured using a Nano-ZS model Zetasizer instrument (mean ± SD, n = 3). The zeta potential of the unmodified SiO_2_ nanoparticles was around -100 mV.

**M6. QCM-D measurement for quantifying polymers and peptide**

**Table S3** Variations in frequency and mass of the polymer coatings detected by QCM-D on SiO_2_ chips.

| **Samples** | -∆F (Hz) | ∆m (ng/cm^2^) |
| --- | --- | --- |
| PEI/PSO_3_ | 1620 ± 97 | 9439 ± 572 |


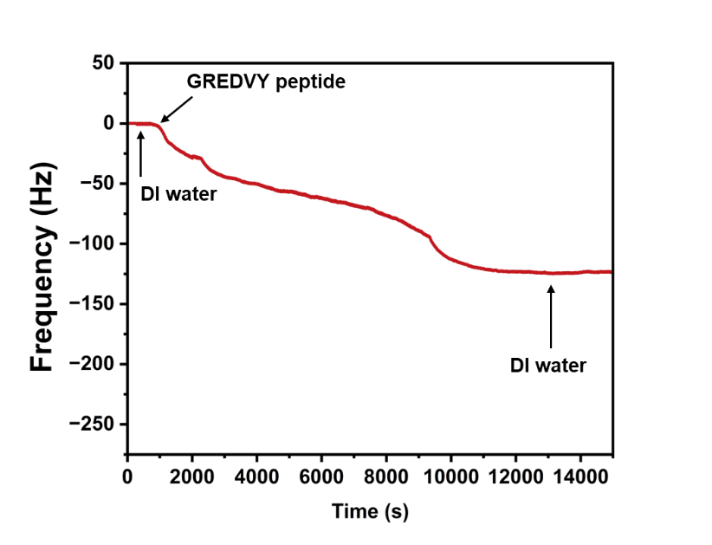


**Figure S4**. Frequency changes measured by QCM-D after the introduction of the GREDVY peptide.

**M7. XPS survey spectra and chemical compositions of different samples**

**
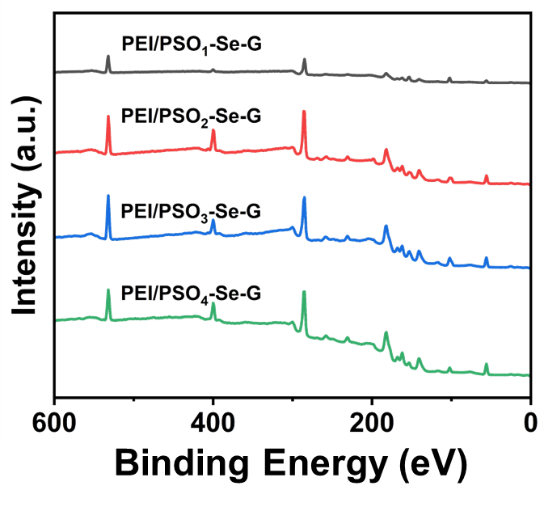
**

**Figure S5.** XPS survey spectra of different surfaces.

**Table S4** The chemical compositions of different samples detected by XPS

| **Surface** | **Elemental composition (At %)** | | | | |  | Se/C |
| --- | --- | --- | --- | --- | --- | --- | --- |
|  | C | N | O | Si | S | Se |  |
| PEI/PSO_1_-Se-G | 48.03 | 5.42 | 18.83 | 11.88 | 12.61 | 3.23 | 0.067 |
| PEI/PSO_2_-Se-G | 49.31 | 14.23 | 13.14 | 6.13 | 13.72 | 3.47 | 0.070 |
| PEI/PSO_3_-Se-G | 48.12 | 9.96 | 14.73 | 8.72 | 14.26 | 4.21 | 0.087 |
| PEI/PSO_4_-Se-G | 48.38 | 11.23 | 10.99 | 4.09 | 20.55 | 4.76 | 0.098 |

**M8. NO release of PEI/PSS-Se samples**


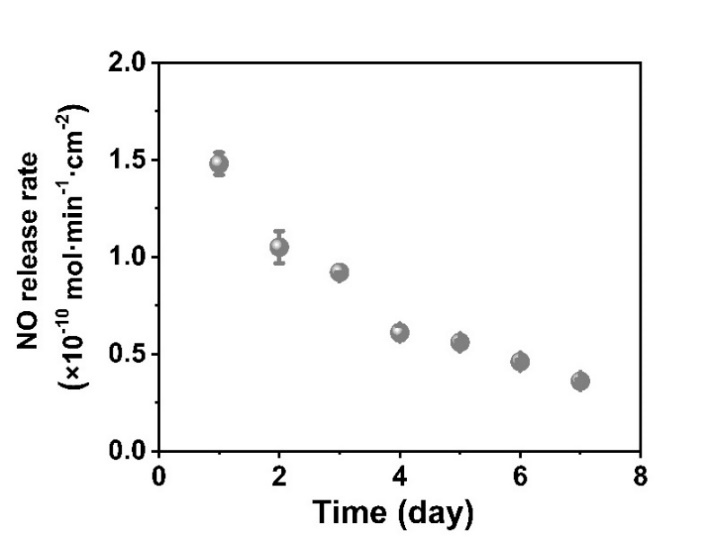


**Figure S6.** No release rates of the PEI/PSS-Se samples within a period of 7 days. No release rate of Si was approximately 0.065 ×10^-10^ mol∙min^-1^∙cm^-2^ (mean ± SD, n = 3).

**M9. HUVSMCs cultured on PEI/PSS-Se**

HUVSMCs were cultured on Si and PEI/PSS-Se samples for 24 h and 72 h. NO donors (65 μM GSNO and 30 μM GSH) were added every 6 h during culture. Fluorescence images are shown in **Figure S7**.


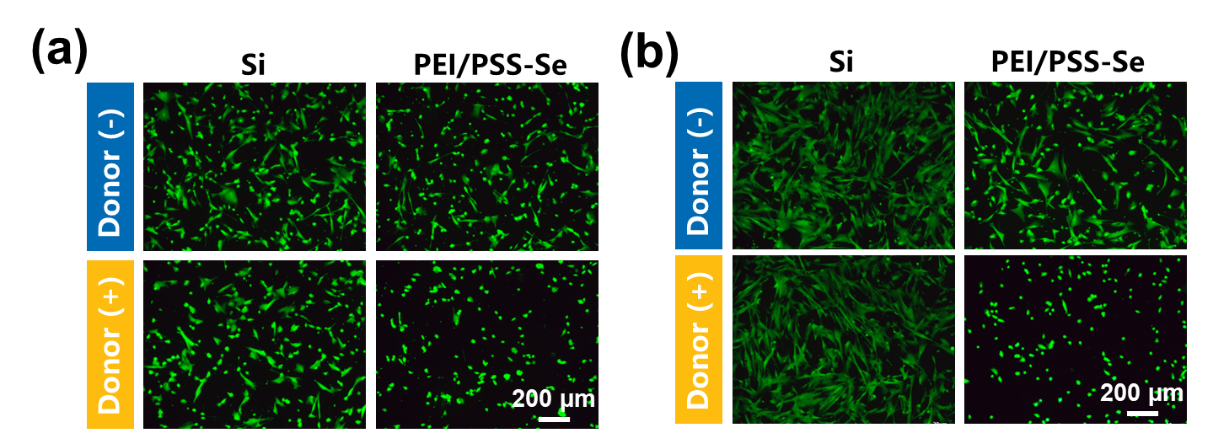


**Figure S7.** Fluorescence images of HUVSMCs on different samples after 24 h (a) and 72 h (b) of incubation.

**M10. Cell viability of HUVSMCs and HUVECs**

HUVSMCs cultured on different samples were stained with calcein-AM and PI to characterize cell viability after 24 h and 72 h of incubation. NO donors (65 μM GSNO and 30 μM GSH) were added every 6 h during the incubation. Fluorescence images and HUVSMC viability are shown in **Figure S8**.


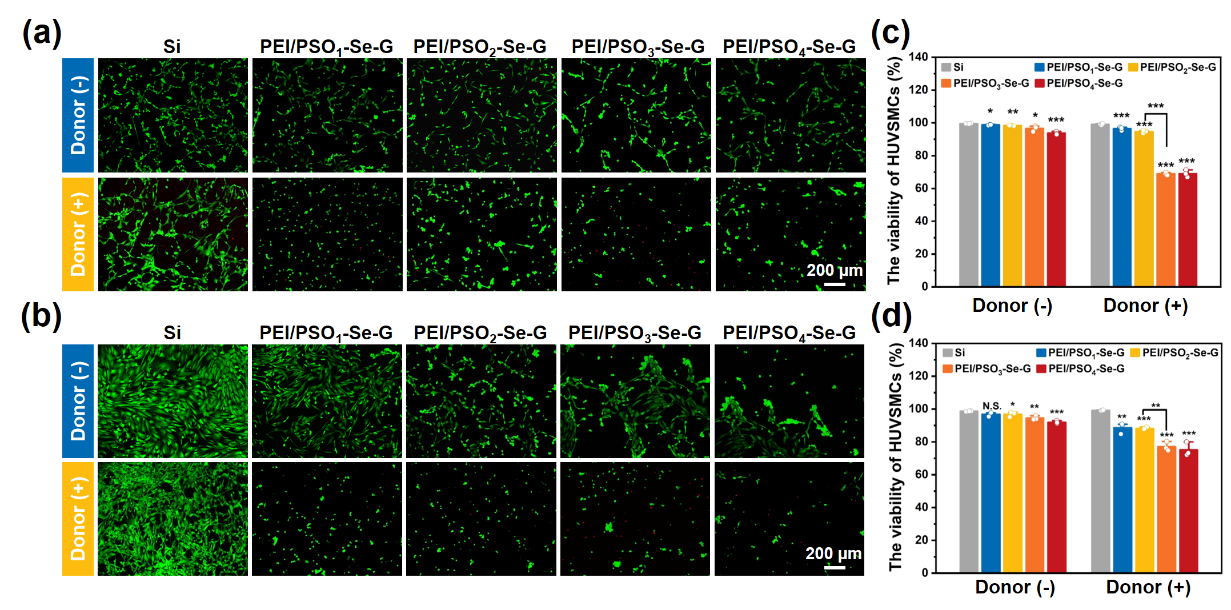


**Figure S8.** Fluorescence images of HUVSMCs on different samples after 24 h (a) and 72 h (b) of incubation (green: calcein-AM, red: PI). Cell viability of HUVSMCs on different samples after 24 h (c) and 72 h (d) of incubation.

HUVECs cultured on different samples were stained with calcein-AM and PI to characterize cell viability after 24 h and 72 h of incubation. NO donors (65 μM GSNO and 30 μM GSH) were added every 6 h during the incubation. Fluorescence images and HUVEC viability are shown in **Figure S9**.


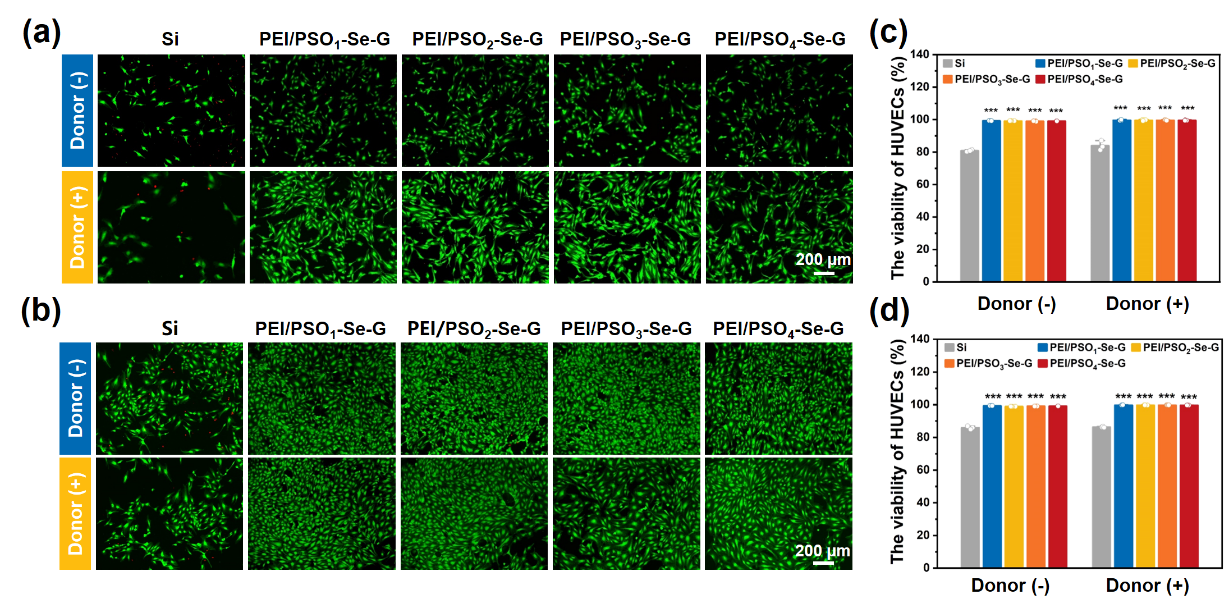


**Figure S9.** Fluorescence images of HUVECs on different samples after 24 h (a) and 72 h (b) of incubation (green: calcein-AM, red: PI). Cell viability of HUVECs on different samples after 24 h (c) and 72 h (d) of incubation.

**M11. HUVECs cultured on PEI/PSO_n_-Se**

Fluorescence images of HUVECs on PEI/PSO_n_-Se surfaces after 24 h and 72 h of incubation are shown in **Figure S10**. NO donors (65 μM GSNO and 30 μM GSH) were added every 6 h during the incubation.


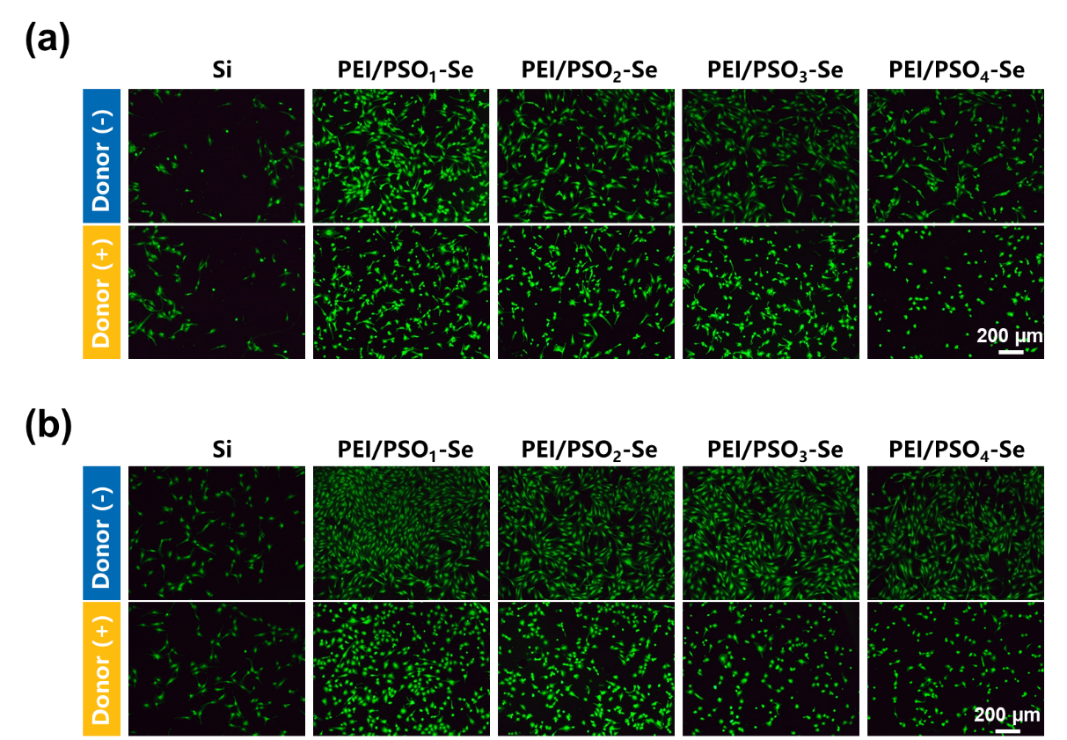


**Figure S10.** Fluorescence images of HUVECs on different samples after 24 h (a) and 72 h (b) of incubation.
