# Supplementary material for: Balancing functions of antifouling, nitric oxide release and vascular cell selectivity for enhanced endothelialization of assembled multilayers
Source: Regen Biomater. 2024 Aug 24;11:rbae096. doi: 10.1093/rb/rbae096 (PMC11422184; doi:10.1093/rb/rbae096)
Supplement: rbae096_Supplementary_Data [file rbae096_supplementary_data.zip › Response to Reviewers.docx]

**Response** **to** **Editor**

1. Meanwhile, the authors should revise the graphic abstract (GA). The current size of your graphic abstract (GA) is incorrect. It should be 3.6 cm*8.4 cm according to the author guideline of Regenerative Biomaterials. Also, the font size in GA should at least be 8 pt. Do NOT enlarge your GA to make letters distinguishable. The canvas itself should be 3.6 cm*8.4 cm and do NOT leave empty margin on either side of GA. Please show the GA immediately following the abstract in the submitted manuscript.

**Response**

Thank you very much for your careful comment. As suggested, we have adjusted the size of the GA and confirmed that all the text is legible at this size. The GA is shown immediately following the abstract in the submitted manuscript.

**Response to reviewer comments**

**Reviewer: 1**

**Comments:**

In this paper, the authors proposed a multifunctional surface construction strategy in the manuscript. Overall, this strategy is clear and reasonable. However, there were still some points that need to be modified or explained before publication. Some comments as follows.

**Response**

We are grateful to the reviewer for the profound and thorough review as well as the positive and encouraging comments.

**Comment 1**

The thickness of the multilayers increased as the assembly process proceeded. Finally, 7.5 bilayers were determined. I think, for better understanding, this decision should be explained.

**Response 1**

Thank you very much for your suggestion. After constructing multilayers by layer-by-layer self-assembly, SeCA was grafted onto the surface to catalytically release NO by decomposing the donor. To determine the optimal number of bilayers for SeCA grafting, samples with 5.5 bilayers, 7.5 bilayers, and 10.5 bilayers were chosen for the preliminary experiments. Healthy HUVECs sustainably generate NO at a level of (0.5-4) × 10^-10^ mol·min^-1^·cm^-2^ to maintain vascular health (Biomaterials 2020, 248, 119981). The NO release rates after the grafting of SeCA are shown in **Figure R1a**. The release rates for samples with 5.5 bilayers were (0.08-0.82) ×10^-10^ mol·min^-1^·cm^-2^ within 4 days. The release rates for samples with 7.5 bilayers and 10.5 bilayers were in the range of (0.56-1.77) ×10^-10^ mol·min^-1^·cm^-2^ within 4 days. There was no significant difference between samples with 7.5 bilayers and those with 10.5 bilayers. Considering that the surfaces constructed with 7.5 bilayers and 10.5 bilayers had higher NO release rates and long-term effects than the surfaces constructed with 5.5 bilayers, the behavior of HUVECs on surfaces constructed with 7.5 bilayers and 10.5 bilayers was studied. As shown in **Figure R1b**, after 48 h of incubation with the donor, the density of HUVECs on surfaces with 7.5 bilayers was higher than that on surfaces with 10.5 bilayers. Therefore, 7.5 bilayers were selected for further studies.

Figure R1. (a) NO release rates of the PEI/PSS-Se samples with different bilayers within 4 days. The NO release rate of Si was ~ 0.065 ×10^-10^ mol∙min^-1^∙cm^-2^. (b) HUVEC density on the PEI/PSS-Se samples with different bilayers after 48 h of culture with the donor (n = 3, ***P* < 0.01).

The NO release rate and HUVEC density data have been added to the supporting material. The reason for determining 7.5 bilayers has been added to the revised manuscript (Page 13).

**Comment 2**

For each layer in the assembly process, more evidences or explanations should be provided for the chemical composition changes of the coating. The current structural analysis is insufficient.

**Response 2**

Thank you very much for your constructive comment. As suggested, FTIR and XPS were used to characterize the chemical composition changes of each layer during the assembly process. As shown in **Figure R2a**, when the number of the assembled bilayers was 0.5, 1.5, 2.5, 3.5, 4.5, 5.5, 6.5 and 7.5 with PEI as the outermost layer, an absorption peak at 1643 cm^-1^ was observed on the surface, which was attributed to the bending vibration of the primary amine groups within the PEI structure. As shown in **Figure R2b**, when the number of the assembled bilayers was 1, 2, 3, 4, 5, 6 and 7 with PSO_3_ as the outermost layer, an absorption peak at 1183 cm^-1^ was observed on the surface, which was attributed to the sulfonic acid group in PSO_3_. The chemical composition of the samples was further determined by XPS. As shown in **Table R1**, when the outermost layer was PEI, the N content was approximately 20%, and the S content was less than 1%. When the outermost layer was PSO_3_, the S content increased from 1% to 6%, while the N content decreased significantly. With the process of layer-by-layer self-assembly, the chemical composition of the surface changed.

The FTIR spectra of surfaces with different bilayers and corresponding explanations have been added to the revised manuscript (Page 15). The elemental composition determined by XPS has been added to the supporting material.

Figure R2. FTIR spectra of surfaces with different numbers of bilayers. (a) The outermost layer was PEI. (b) The outermost layer was PSO_3_.

**Table R1** The chemical compositions of different samples detected by XPS

(N.D. for non-detectable)

| **Bilayers** | **Elemental composition (%)** | | | | |
| --- | --- | --- | --- | --- | --- |
|  | C | Si | O | N | S |
| 0.5 | 65.30 | 0.90 | 10.13 | 23.67 | N.D. |
| 1 | 63.52 | 1.13 | 22.37 | 7.44 | 5.54 |
| 1.5 | 53.04 | 9.09 | 22.20 | 15.67 | N.D. |
| 2 | 59.88 | 5.09 | 22.41 | 7.01 | 5.61 |
| 2.5 | 61.14 | 3.88 | 10.70 | 23.08 | 1.20 |
| 3 | 63.52 | 1.52 | 22.10 | 7.27 | 5.59 |
| 3.5 | 68.93 | 1.28 | 4.93 | 24.38 | 0.47 |
| 4 | 64.12 | 0.73 | 21.20 | 8.06 | 5.90 |
| 4.5 | 62.79 | 3.15 | 10.11 | 23.00 | 0.96 |
| 5 | 64.20 | N.D. | 22.14 | 7.91 | 5.74 |
| 5.5 | 61.12 | 4.53 | 12.45 | 20.95 | 0.94 |
| 6 | 64.47 | 0.68 | 21.95 | 7.12 | 5.79 |
| 6.5 | 65.14 | 1.89 | 17.26 | 15.72 | N.D. |
| 7 | 64.40 | N.D. | 22.24 | 7.38 | 5.98 |
| 7.5 | 61.12 | 4.53 | 12.45 | 20.96 | 0.94 |

**Comment 3**

For example, hydroxyl concentration on the substrate surface before and after treatment with piranha wash solution.

**Response 3**

As suggested, water contact angle, FTIR and XPS were used to characterize the hydroxyl groups before and after treatment with piranha solution. As shown in **Figure R3a**, the water contact angle of the Si surface was ~60°. After treatment with piranha solution, the water contact angle of the Si-OH surface was ~5°, indicating superhydrophilicity. A wide absorption band in the range of 3600-3400 cm^-1^ was observed on the Si-OH surface (**Figure R3b**), indicating the presence of hydroxyl groups. As shown in **Table R2**, the ratios of O/Si on the Si and Si-OH surfaces were 0.66 and 10.54, respectively. The significant increase in O/Si on the Si-OH surface compared to that on the Si surface indicated that a large number of hydroxyl groups appeared on the surface after the piranha solution treatment (Colloids Surf., B, 2014, 123, 181).

Since hydroxyl groups on silicon substrates are prone to oxidation and contamination (Mater. Sci. Semicond. Process. 2014, 26, 25), it is difficult to determine the exact hydroxyl concentration on the substrate surface. We will carefully take this into consideration in future studies and hope that the reviewer will be understanding.

Figure R3. The water contact angle (a) and FTIR spectra (b) of Si and Si-OH surfaces.

**Table R2** The chemical composition of Si and Si-OH surfaces detected by XPS

| **Surfaces** | **Elemental composition (%)** | | | O/Si |
| --- | --- | --- | --- | --- |
|  | C | Si | O |  |
| Si | 16.46 | 50.08 | 33.46 | 0.66 |
| Si-OH | 63.79 | 1.33 | 14.02 | 10.54 |

The water contact angle and FTIR spectra before and after piranha solution treatment have been added to the revised manuscript (Page 16). The chemical compositions determined by XPS have been added to the supporting material.

**Comment 4**

Have the authors considered stability experiments for the coatings? This result has important guiding significance for future application directions.

**Response 4**

Thank you very much for your constructive comment. As suggested, the stability of the coating was characterized by UV‒Vis spectrophotometer and spectroscopic ellipsometer. Specifically, the PEI/PSO_3_-Se-G samples were incubated in PBS solution at 37 °C for 7 days with shaking at 100 rpm. As shown in **Figure R4a**, after soaking the PEI/PSO_3_-Se-G samples for 7 days, the absorbance of the PBS solution before and after soaking the sample was basically unchanged. The thicknesses of the PEI/PSO_3_-Se-G samples did not obviously differ before and after 7 days of incubation (**Figure R4b)**. These data indicated the stability of the surface coating.

The corresponding data and discussion have been added to the revised manuscript (Page 18 and Page 19).

Figure R4. Stability of the surface coating. (a) Absorbance of the PBS solution before and after soaking the PEI/PSO_3_-Se-G samples for 7 days. (b) Thickness of the PEI/PSO_3_-Se-G samples before and after 7 days of incubation in PBS solution.

**Comment 5**

I'm not sure if there were any errors in using the images. Figure 6 and Figure 7 seem to be the same. The repeated use of images should be prohibited.

**Response 5**

Thank you very much for your careful review. As suggested, **Figure 6** has been changed to the correct image (Page 22, Figure 6). We have thoroughly examined all the images to guarantee accuracy and avoid any potential errors in their usage.

**Comment 6**

Table 1 reappears on the last page. To ensure the quality of the final paper, this phenomenon needs to be verified.

**Response 6**

Thank you very much for your careful review. In the revised manuscript, it has been confirmed that Table 1 will not be redundantly included.

**Reviewer: 2**

**Response**

Thank you for your positive comments.

**Comment 1**

In the method section, it is essential to specify the molecular weight of the dialysis bags utilized.

**Response 1**

Thank you very much for your suggestion. The molecular weight of dialysis bags utilized in method section was 3500 Da. As suggested, we have added the molecular weight of the dialysis bags to the revised manuscript (Page 7).

**Comment 2**

Although the adsorption of GREDVY peptide is described as occurring under weak acidic conditions, there is a lack of clear explanation regarding the force of interaction between the peptide and substrate in the results. Additionally, it would be beneficial to elucidate the stability of the adsorbed peptide.

**Response 2**

Thank you very much for your suggestion. The isoelectric point of GREDVY peptide is 3.6. When the external pH is 5, the peptide is negatively charged, thus binding to the positively charged PEI on the surface through electrostatic interactions. The explanations have been added to the revised manuscript (Page 17).

As suggested, the stability of the absorbed peptide was tested by UV-Vis spectrophotometer, spectroscopic ellipsometer and FTIR. The PEI/PSO_3_-Se-G samples were incubated in PBS solution at 37 °C for 7 days with shaking at 100 rpm. As shown in **Figure R5a**, the absorbance of the PBS before and after soaking the PEI/PSO_3_-Se-G samples was basically unchanged. The thicknesses of the PEI/PSO_3_-Se-G samples did not obviously differ before and after 7 days of incubation (**Figure R5b**). As shown in **Figure R5c**, after 7 days of incubation in PBS at 37 °C, the absorption peaks at 1630 cm^-1^, attributed to -C=NH and 1190 cm^-1^, attributed to -C-OH were still present on the PEI/PSO_3_-Se-G surface, indicating that the peptide was still on the surface.

The relevant data and the corresponding discussion have been added to the revised manuscript (Page 18 and Page 19).

Figure R5. (a) Absorbance of the PBS solution before and after soaking the PEI/PSO_3_-Se-G samples for 7 days. Thicknesses (b) and FTIR spectra (c) of the PEI/PSO_3_-Se-G samples before and after 7 days of incubation in PBS solution.

**Comment 3**

The coupling of molecules containing carboxyl and amino groups was achieved using EDC; however, both PEI and selenocysteine solely possess amino groups without carboxyl groups. Therefore, it remains unclear how this coupling was accomplished.

**Response 3**

Thank you very much for your professional review. In this work, the coupling of PEI and selenocysteine was not achieved by EDC but by *N, N'*−carbonyl diimidazole. The mechanism of coupling PEI and selenocysteine by *N,* *N'*−carbonyl diimidazole is shown in **Scheme R1**. The method used to couple amino groups by *N, N'*−carbonyl diimidazole was reported previously (*J. Mater. Chem. B,* 2017, 5, 980. *Macromolecules*, 2004, 37, 9418). Upon the addition of *N,* *N'*−carbonyl diimidazole solution to the selenocysteine solution, the amino group of selenocysteine reacts with the imidazole group of *N, N'*−carbonyl diimidazole to form an intermediate, which then continues to react with the amino group of PEI on the surface. The relevant explanations have been added to the revised manuscript (Page 7).

Scheme R1. Coupling PEI and selenocysteine by *N, N'*−carbonyl diimidazole.

**Comment 4**

While there are characterization groups for PEI/PSOn-Se and PEI/PSOn-Se-G materials, only PEI/PSOn-Se-G served as experimental groups in biological experiments with Si as a control group. This omission raises questions about proving whether G has an impact on cell adhesion, proliferation, migration, etc., since no data from PEI/PSOn-Se group were provided.

**Response 4**

Thank you very much for your careful review. As suggested, fluorescence images of HUVECs on PEI/PSO_n_-Se surfaces are shown in **Figure R6**. The PEI/PSO_n_-Se surface did not significantly promote HUVEC proliferation when the donor was added. Therefore, the GREDVY peptide was designed to selectively promote HUVEC proliferation. Compared with those on PEI/PSO_n_-Se surfaces, HUVECs on PEI/PSO_n_-Se-G surfaces exhibited improved adhesion and proliferation, demonstrating the efficacy of the GREDVY peptide in enhancing HUVEC attachment and growth.

The fluorescence images of HUVECs on PEI/PSO_n_-Se surfaces have been added to the supporting material.

Figure R6. Fluorescence images of HUVECs on the different samples after 24 h (a) and 72 h (b) of incubation.

**Comment 5**

Utilizing high-resolution Se3d peak height in Figure 5b may not be considered a rigorous approach for demonstrating elemental abundance; instead, obtaining atomic ratios based on SURVEY spectrum would provide more reliable evidence.

**Response 5**

Thank you very much for your professional review. As suggested, the PEI/PSO_n_-Se-G surface was characterized by XPS. **Figure R7** shows the XPS survey spectra of the different surfaces. **Table R3** shows elemental compositions measured by XPS. The ratios of Se/C on the PEI/PSO_1_-Se-G, PEI/PSO_2_-Se-G, PEI/PSO_3_-Se-G, and PEI/PSO_4_-Se-G surfaces were 0.067, 0.070, 0.087, and 0.098, respectively. The gradual increase in atomic ratios of Se/C indicates a progressive increase in the Se content across the various surfaces.

Figure R7. XPS survey spectra of different surfaces.

**Table R3** The chemical compositions of different surfaces detected by XPS

| **Surfaces** | **Elemental composition (At %)** | | | | |  | Se/C |
| --- | --- | --- | --- | --- | --- | --- | --- |
|  | C | N | O | Si | S | Se |  |
| PEI/PSO_1_-Se-G | 48.03 | 5.42 | 18.83 | 11.88 | 12.61 | 3.23 | 0.067 |
| PEI/PSO_2_-Se-G | 49.31 | 14.23 | 13.14 | 6.13 | 13.72 | 3.47 | 0.070 |
| PEI/PSO_3_-Se-G | 48.12 | 9.96 | 14.73 | 8.72 | 14.26 | 4.21 | 0.087 |
| PEI/PSO_4_-Se-G | 48.38 | 11.23 | 10.99 | 4.09 | 20.55 | 4.76 | 0.098 |

The ratios of Se/C and the corresponding discussion have been added to the revised manuscript (Page 20). XPS survey spectra and elemental compositions of different surfaces have been added to the supporting material.

**Comment 6**

Although this designed coating aims to be applied on vascular interventional devices, silicon materials are rarely used in such devices compared to more representative metal materials like stainless steel. It would be helpful if authors could explain their rationale behind choosing silicon over other commonly employed metals.

**Response 6**

Thank you very much for your professional suggestion. The silicon wafer was used as a model substrate because of its ease of subsequent surface characterization. Considering the advantages of layer-by-layer self-assembly technology for the substrate universality, this designed coating strategy is expected to be applied for the surface modification of metal substrates such as stainless steel. In our work, the surface modification strategy was further extended to SiO_2_ nanoparticles. The zeta potential of these modified SiO_2_ nanoparticles, observed during the layer-by-layer self-assembly process of multilayers, is depicted in **Figure R8**.

As suggested, the reason for choosing silicon wafers has been added to the revised manuscript (Page 13). Thank you once again for your valuable insights. We plan to conduct further research on representative materials, such as stainless steel, in our upcoming studies.

Figure R8. The zeta potential of the modified SiO_2_ nanoparticles in ultrapure water measured using a Nano-ZS model Zetasizer instrument (mean ± SD, n = 3).

**Reviewer: 3**

**Comments**

The paper submitted by Zhang et al. investigates the endothelialization induced by self-assembled multilayers based on one copolymer, one polymer, organic selenium and a peptide. The manuscript is clear, well written and the conclusions are supported by the results. However, some corrections are needed before publication:

**Response**

We are grateful to the reviewer for the profound and thorough review as well as the positive and encouraging comments.

**Comment 1**

In the method section, it is essential to specify the molecular weight of the dialysis bags utilized.

**Response 1**

Thank you very much for your professional comments. The molecular weight of the dialysis bags utilized was 3500 Da. As suggested, we have added the molecular weight of the dialysis bags to the revised manuscript (Page 7).

**Comment 2**

GPC spectra must be provided. It seems that dispersity values are quite low for a classical radical polymerization.

**Response 2**

Thank you very much for your professional comments. During the layer-by-layer self-assembly process, the molecular weights of the two polyelectrolytes may affect the constructed multilayers (*Langmuir* 2003, 19, 2491). Therefore, dialysis bags with a molecular weight of 8000 Da were used to remove low molecular weight products. Therefore, the polydispersity values are lower than those of classical radical polymerization.

Dialysis bags with a molecular weight of 3500 Da are commonly used for polymer purification in classical radical polymerization (*ACS Appl. Mater. Interfaces* 2023, 15, 35860). Accordingly, the PSO_n_ copolymers purified using dialysis bags with a molecular weight of 3500 Da were characterized by GPC, and the resulting spectra are presented in **Figure R9**. The molecular weights of the PSO_n_ copolymers synthesized with different feed ratios ranged from (1.3-1.4) ×10^4^ g/mol, and the polydispersity indices (Đ) of the copolymers ranged from 1.21 to 1.22. There were no significant differences in the molecular weights or Đ values of the PSO_n_ copolymers.

As suggested, GPC spectra have been added to the supporting material.

Figure R9. The GPC spectra of different copolymers.

**Comment 3**

Which was the conversion percentage?

**Response 3**

Thank you very much for your professional comments. The conversion percentages ranged from 86% to 87% for the different copolymers. These data have been added to the revised manuscript (Page 12)

**Comment 4**

The discussion of the results is quite scarce and should be completed with some literature references?

**Response 4**

Thank you very much for your professional comments. As suggested, we have added additional discussions and relevant literature to deepen the analysis of our results in the revised manuscript (Page 11, Page 12, Page 13, Page 14, Page 15, Page 18, Page 19, Page 20, Page 26, Page 27, and Page 31).

**Comment 5**

Which solvent was used for the GPC? at which solvent rate, temperature, which stardards?

**Response 5**

Thank you very much for your professional comments. The molecular weights (*M*_n_, Da) and polydispersity indices (Đ) of copolymers were determined using gel permeation chromatography (GPC) with water containing 0.2 M NaNO_3_ and 0.1 M NaH_2_PO_4_ and adjusted to pH = 9 at 30 °C (flow rate: 1 mL/min). GPC was calibrated based on the narrow molecular weight distribution of PEG standards.

As suggested, these details about the GPC test have been added in the revised manuscript (Page 8).

**Comment 6**

For the NMR calculations, the authors must indicate which peaks they have used as characteristic peaks for PSS and POEGMA.

**Response 6**

Thank you very much for your professional comments. The chemical structure of the PSO_n_ copolymers is shown in **Figure R10a**. **Figure R10b** shows typical ^1^H-NMR spectra of the synthesized copolymers. The characteristic peaks with a chemical shift at 3.0-3.6 ppm belong to the methylene proton on the side chain of the POEGMA fraction, and the characteristic peaks with a chemical shift at 6.0-8.1 ppm belong to the proton on the benzene ring of the PSS fraction. The contents of POEGMA and PSS in the PSO_n_ copolymers were calculated according to the integrated area of these two groups of characteristic proton peaks.

As suggested, we have added detailed explanations for the calculations of PSS and POEGMA contents in the revised manuscript (Page 12).

Figure R10. Chemical structure (a) and ^1^H−NMR spectra using heavy water (D_2_O) as the solvent (b) of the PSO_n_ copolymers.

**Reviewer: 4**

**Comments**

The manuscript addresses a significant issue in biomaterials science, specifically improving hemocompatibility through multifunctional surface modifications. The authors have made commendable efforts to balance multiple surface functions, which is a challenging task. Some revisions should be addressed before its publication.

**Response**

We are grateful to the reviewer for the profound and thorough review as well as the positive and encouraging comments.

**Comment 1**

Since PEI is positively charged and very fouling, how did the authors make the films low-fouling when PEI is at the last layer?

**Response 1**

Thank you very much for your in-depth review. We completely agree with your statement that PEI is positively charged and very fouling. In our study, we constructed the multilayers by alternatively depositing PEI and PSO_n_. There was an anti-fouling content POEGMA in the multilayers. As reported previously, films constructed by layer-by-layer technology could be intermixed and polymer chains can diffuse in the multilayers (Macromolecules 2021, 54, 9372). Although the last layer was PEI, the POEGMA component, PSS component, NO and peptide on the surface also played a role. Compared to the PEI/PSS-Se surface, the number of HUVSMCs on the PEI/PSO_4_-Se-G surface was lower without the NO donor (**Figure R11**). Upon the addition of the NO donor, the PEI/PSO_4_-Se-G surface resulted in a better cell-resistance effect, indicating the property of the modified surface was governed by the synergistic effects of all the incorporated components.

Figure R11. HUVSMC morphology on different samples after 24 h (a) and 72 h (b) of incubation.

**Comment 2**

How about the zeta potential after each layer coating? It is suggested to do the coatings on spherical particles to monitor the zeta potential.

**Response 2**

Thank you very much for your professional comments. As suggested, we chose SiO_2_ nanoparticles to measure the zeta potential after each layer coating. As shown in **Figure R12**, the zeta potential of the unmodified SiO_2_ nanoparticles was around -100 mV. When the outermost layer was PEI, the particles were positively charged and the zeta potential was approximately 15~50 mV. When the outermost layer was PSO_3_, the particles were negatively charged and the zeta potential was approximately -60~-40 mV.

The zeta potentials measured for the SiO_2_ nanoparticles have been added to the supporting material and the corresponding discussion has been added to the revised manuscript (Page 15 and Page 16).

Figure R12. The zeta potential of the modified SiO_2_ nanoparticles in ultrapure water measured using a Nano-ZS model Zetasizer instrument (mean ± SD, n = 3). The zeta potential of the unmodified SiO_2_ nanoparticles was approximately -100 mV.

**Comment 3**

QCM measurements are recommended to check the layer coatings and quantify the deposition of polymers and peptide.

**Response 3**

Thank you very much for your professional comments. As suggested, multilayers constructed by layer-by-layer technology were measured by QCM-D. As shown in **Table R4**, after constructing 7.5 bilayers on SiO_2_ chips, the resonance frequency of the chip decreased by ~1620 Hz. According to the Sauerbrey equation, the mass of chips increased by ~9439 ng/cm^2^ after 7.5 bilayers were constructed by PEI and PSO_3_.

**Table R4**. Variations in frequency and mass after constructing 7.5 bilayers on SiO_2_ chips measured by QCM-D

| Samples | -∆F (Hz) | ∆m (ng/cm^2^) |
| --- | --- | --- |
| PEI/PSO_3_ | 1620 ± 97 | 9439 ± 572 |

As shown in **Figure R13**, after introducing the GREDVY peptide, the resonance frequency of the SiO_2_ chip decreased by ~130 Hz. According to the Sauerbrey equation, the mass of the chips increased by ~766 ng/cm^2^ after the introduction of the GREDVY peptide on the chips.

Figure R13. Frequency changes measured by QCM-D after the introduction of the GREDVY peptide.

These data and corresponding discussion have been added to the supporting material and revised manuscript (Page 16 and Page 17).

**Comment 4**

The NO release rates are discussed, but more detailed kinetic studies would strengthen the conclusion. Perform time-course experiments to measure NO release at multiple time points and provide a kinetic model.

**Response 4**

Thank you very much for your professional comments. Time-course experiments were performed to measure the NO release rate and cumulative release amount of NO catalyzed by different samples. As shown in **Figure R14a**, over time, the NO release rate gradually decreased. The release profile of NO was plotted for four samples in terms of the cumulative release amount as shown in **Figure R14b**. The NO release at multiple time points of the different samples followed a logarithmic model; the kinetic equation can be described as y = A ln(x) + B. A similar phenomenon was observed in a previous report (*Macromol. Mater. Eng*., 2022, 307, 2100862).

The kinetic model and corresponding discussion have been added to the revised manuscript (Page 19) and supporting material.

Figure R14. (a) NO release rate of different samples. (b) Cumulative release amount of NO catalyzed by different samples (mean ± SD, n = 3).

**Comment 5**

The increase in cGMP concentration is significant, but more detailed mechanistic studies are needed. It could be good to perform Western blotting or qPCR to measure the expression levels of key signaling molecules involved in the NO-cGMP pathway.

**Response 5**

Thank you very much for your professional comments. Just as you said, in the NO-cGMP pathway, α-SMA is a key signaling molecule for controlling HUVSMCs with a typical contractile phenotype. After 72 h of incubation of HUVSMCs with PEI/PSO_3_-Se-G samples, the expression level of α-SMA in HUVSMCs was tested by Western blotting. As shown in **Figure R15a**, GAPDH was used as a reference protein. The addition of donors did not significantly affect the expression level of GAPDH in HUVSMCs. Compared with HUVSMCs cultured without donors, HUVSMCs cultured with donors exhibited upregulated α-SMA expression (**Figure R15b**). The PEI/PSO_3_-Se-G samples specifically suppressed the overgrowth of HUVSMCs, and maintained their contractile phenotype by upregulating the expression of α-SMA in the NO-cGMP pathway. The Western blotting data and corresponding discussion have been added to the revised manuscript (Page 10, Page 26 and Page 27).

Figure R15. Western blotting analysis of α-SMA in the HUVSMCs on PEI/PSO_3_-Se-G samples after 72 h of incubation. (a) GAPDH, (b) α-SMA.

**Comment 6**

The promotion of HUVEC adhesion and proliferation is promising. To strengthen this section, please perform additional experiments to measure endothelial marker expression (e.g., CD31, VE-cadherin) using flow cytometry or immunofluorescence.

**Response 6**

Thank you very much for your professional comments. As suggested, immunofluorescence staining was performed to detect the expression of an endothelial specific marker (CD31) in HUVECs. After 24 h of incubation, the immunofluorescence intensity of CD31 in HUVECs cultured on the PEI/PSO_3_-Se-G sample was stronger compared to that on the Si control sample (**Figure R16a**). The addition of donors did not significantly affect the expression level of CD31 in HUVECs.

HUVECs cultured on different samples for 72 h are shown in **Figure R16b**. The CD31 immunofluorescence intensity of HUVECs on the Si and PEI/PSO_3_-Se-G samples increased significantly compared with that after 24 h of incubation. After 72 h of incubation without the donor, HUVECs spread to form a monolayer with strong immunofluorescence intensity of CD31 on the PEI/PSO_3_-Se-G samples. The addition of donors did not significantly affect the expression level of CD31 in HUVECs.

The data suggested that compared with the Si sample, the PEI/PSO_3_-Se-G sample significantly promoted HUVECs proliferation. This trend is in accordance with the results for the adhesion and proliferation of HUVECs. The immunofluorescence data and corresponding discussion have been added to the revised manuscript (Page 10 and Page 31).

Figure R16. Immunofluorescence images of HUVECs on different samples after 24 h (a) and 72 h (b) of incubation (red: CD31, blue: DAPI).
